# Deciphering the Regulatory Circuits of RA3 Replication Module - Mechanisms of the Copy Number Control

**DOI:** 10.3390/ijms23179964

**Published:** 2022-09-01

**Authors:** Aleksandra Markowska-Barkic, Ewa Lewicka, Magdalena Czeredys, Monika Mitura, Grazyna Jagura-Burdzy

**Affiliations:** Laboratory of DNA Segregation and Life Cycle of Proteobacteria, Institute of Biochemistry and Biophysics, Polish Academy of Sciences, Pawinskiego 5a, 02-106 Warsaw, Poland

**Keywords:** plasmid replication, RA3, IncU, RepA repressor, antisense RNA, transcriptional activation

## Abstract

The RA3 plasmid, the archetype of IncU incompatibility group, represents a mosaic-modular genome of 45.9 kb. The replication module encompasses *repA* and *repB* (initiator) surrounded by two long repetitive sequences DR1 and DR2 of unknown function. Here, we mapped the origin of replication *oriV* to the 3′ end of *repB* and showed that *oriV* was activated by the transcription coming from *orf02revp* in the adjacent stability module. Using various in vivo and in vitro methods we demonstrated that the *repB* expression proceeded either from *repBp* located in the intergenic *repA-repB* region or from the upstream strong *repAp* that was autoregulated by RepA. Additionally, the *repBp* activity was modulated by the transcription from the overlapping, divergently oriented *repXp*. Both *repX*mRNA (antisense for *repA*mRNA) and its small polypeptide product, RepX, were strong incompatibility determinants. Hence, we showed that the sophisticated RA3 copy number control combined the multivalent regulation of *repB* expression, RepB titration by DR1, and transcriptional activation of *oriV*, dependent on the RA3 global regulatory network. Similarly organized replicons have been found in diverse bacterial species confirming the significance of these mechanisms in establishing the IncU plasmids in a broad spectrum of hosts.

## 1. Introduction

Bacterial plasmids, the basic components of the mobile gene pool, are vital vehicles for metabolic traits, pathogenicity determinants, and antibiotics resistance loci improving the adaptation of their hosts to changing environmental conditions. A special role in the horizontal gene pool dissemination is played by the broad-host-range (BHR) conjugative plasmids, capable of transfer to and then replication and stable maintenance in the phylogenetically distant bacterial species [1].

**Figure 1:**
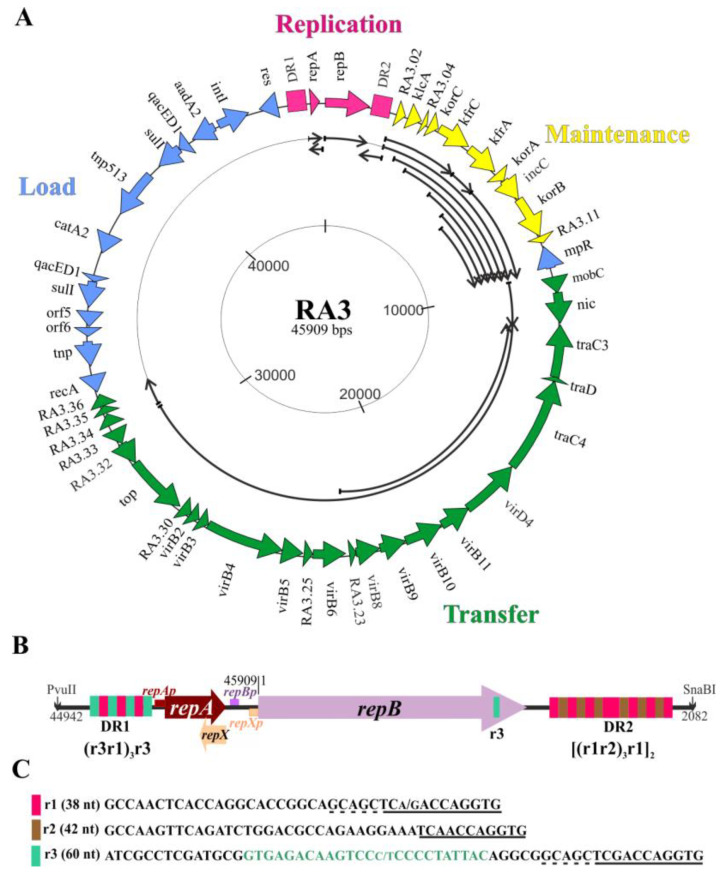
RA3 map (DQ401103). (**A**) Transcriptional organization. The ORFs are indicated by thick arrows pointing out the direction of transcription. Long direct repeats in the replication module are symbolized by rectangulars. Functional modules are labelled with different colors: replication in red, maintenance in yellow, conjugative transfer in green, and load (mainly In10) in blue. Thin arrows in the inner circle demonstrate the experimentally confirmed transcripts from the RA3 backbone. (**B**) Close-up of the replication module between PvuII and SnaBI restriction sites (3039 bp). Note that the annotation of the RA3 genome starts from the ATG codon of *repB* [9]. Promoters identified in the module are indicated by small boxes [9]. Long direct repeats DR1 and DR2 are built of the three repetitive motifs r1, r2, and r3 according to the formulas below the schemes. (**C**) DNA sequences of the repetitive motifs. The sequences conserved in all motifs are underlined. Part of r3 present in the 3′ end of the *repB* is shown in green.

The object of our studies, the RA3 plasmid of 45.9 kb, an archetype of the IncU incompatibility group of *E. coli* (IncP-6 for *Pseudomonas plasmids*) [2] belongs to the BHR conjugative plasmids, conferring resistance to chloramphenicol, streptomycin, and sulfonamides. The IncU representatives are widely spread in the fish hatcheries, fresh water springs, waste factories, and hospitals [3,4,5,6,7,8]. RA3, initially isolated from *Aeromonas hydrophila* [2], can transfer, replicate, and be quite stably maintained in representatives of *Alpha-, Beta-*, and *Gammaproteobacteria* [9,10,11]. It has a modular structure with spatially separated blocks of genes engaged in the replication, stable maintenance, conjugation, and phenotypic traits (Figure 1A). A similar modular structure has been described for many environmental plasmids including members of the PromA group [12]. Although RA3 and the PromA plasmids (e.g., pSB102, pIPO2, pTer331) belong to different incompatibility groups (based on their replication systems), they share the conjugative module distinct from other well characterized promiscuous conjugative systems in terms of content and organization. Both groups also carry genes resembling the maintenance modules of the IncP plasmids.

**Figure 2:**
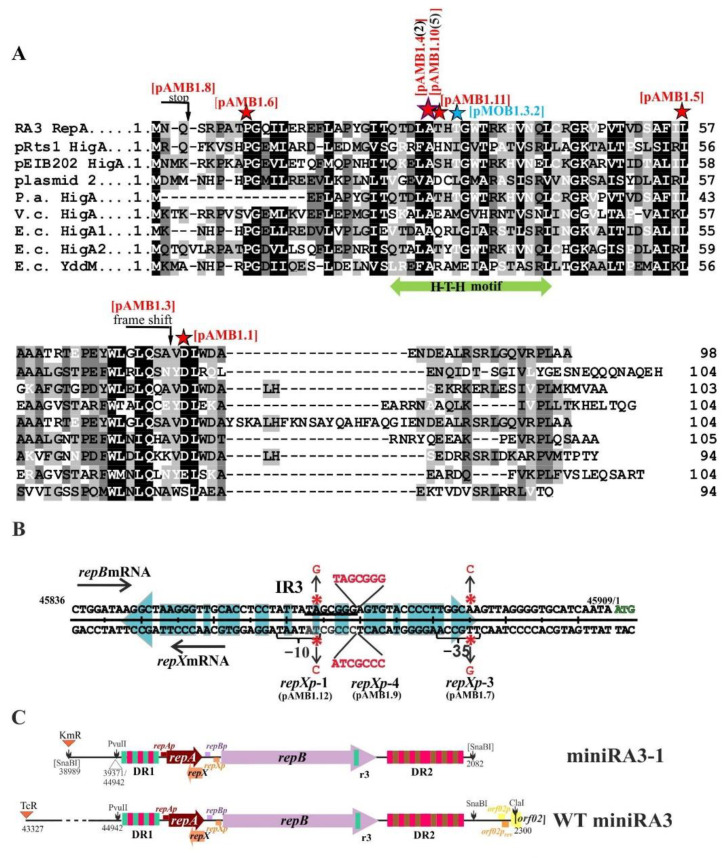
Analysis of the RA3 minireplicon derivatives. (**A**) Comparison of the plasmidic (beyond the IncU group) and chromosomal homologs of the RepA protein (RepA_RA3,_ WP_012477889, HigA from Rts1 of *Proteus vulgaris*, WP_011039854, HigA from pEIB202 of *Edwardsiella tarda*, WP_012850372, ORF of plasmid 2 of *Nitrosomonas eutropha* C91, WP_041353920, HigA of *Pseudomonas aeruginosa* ST1006, WP_238839714; HigA of *Vibrio cholerae* O395, WP_001232701; HigA1 of *Escherichia coli* QH21-5-14, GeneBank MCB8828485; HigA2 of *E. coli* 100063-3, WP_174576884 and YddM of *E. coli* K-12 substr. MG1665, GeneBank AAD13441). The identical/ similar residues in 8 or 9 derivatives are marked in black, in 6 or 7 derivatives are shaded dark grey, and in 3 to 5 in the light grey with black or white lettering. The putative H-T-H motif identified in RepA_RA3_ is shown by green arrow. Asterisks above the RepA_RA3_ sequence point out amino acid substitutions in the RepA derivatives encoded in the mutated minireplicons. The larger asterisk denotes A29 substitutions into V or G that appeared in five and two clones, respectively. Arrows above the sequence mark the RepA truncations. (**B**) The part of the intergenic *repA-repB* region with putative *repXp* motifs oriented divergently toward upstream *repBp*. The coordinate 1 marks the start codon for RepB. The long palindromic sequence (IR3) is shown in blue. The substitutions and insertion detected in the three minireplicon derivatives are indicated in red. The duplicated sequence in the *repXp-*4 mutant is underlined. (**C**) Comparison of the WT miniRA3 boundaries with the miniRA3-1, the representative of the mutated minireplicons. The RA3 coordinates are indicated as well as identified promoter sequences. SnaBI in brackets corresponds to the inactivated SnaBI restriction site by the insertion of Km^R^ cassette during minireplicon variants construction. The pair of coordinates at PvuII site indicates the extent of the internal deletion. The integration of Tc^R^ cassette in RA3 led to the deletion between RA3 coordinates 2300 and 43327 and construction of WT miniRA3 with an introduced ClaI site. The color code of ORFs and DRs follows Figure 1B.

The replication module of RA3 (~3 kb) consists of the *repA* and the *repB* transcribed in the same direction and enclosed between long repetitive sequences: DR1 (339 bp) upstream of the *repA* and DR2 (557 bp) downstream of the *repB* (Figure 1B). The *repA* gene encodes a protein, which is homologous to the *higA* family of antidote proteins for the post-segregational killing systems and contains the H-T-H motif of Cro/cI type repressors (Figure 2A). Significantly no adjacent ORF encoding HigB-like toxin has been identified in RA3. RepB shows homology to putative replication initiation proteins from a wide variety of plasmids of *Alpha-, Beta-,* and *Gammaproteobacteria* as well as *Firmicutes* [9] (Figure S1). The DR regions are composed of repetitive elements r1 (38 bp), r2 (42 bp), and r3 (60 bp), which share 11 up to 16 bp sequences at the 3′ ends (Figure 1C). The DR1 consists of r1 and r3, organized as (r3r1)_3_r3, whereas DR2 is built of r1 and r2 organized as [(r1r2)_3_r1]_2_ [9]. The central 25 bp part of r3 occurs in a single copy in the 3′ end of *repB* ORF. A tandem variant of the r3 sequence has been found in the 3′ end of *rep* gene of Rms149 plasmid [13] of the same incompatibility group (70% identity of RepB proteins). The r3-like sequences occur within *repB* or downstream of *repB* in a single or tandem copies in other IncU plasmids with homologous *repB* genes [13,14,15]. In the preliminary studies on RA3, the *repB* gene and the downstream DR2 repeats seemed to be sufficient for replication in *E. coli polA* strain, suggesting that this region encompassed *oriV* and that *repB* encodes a functional replication initiation protein [9].

Three promoters have been identified in the replication module: one upstream of the *repA* and two within the 172 bp intergenic *repA-repB* region, one weak firing toward *repB* and a divergent, strong *repXp* that hypothetically was responsible for the synthesis of an antisense RNA for *repA*mRNA (Figure 1B). In silico analysis indicated that the antisense mRNA might encode a polypeptide of 38 amino acids designated RepX (Figure S2). To decipher the regulatory circuits present in the replication module, in vivo and in vitro transcriptional analysis along with genetic studies were initiated. Here, we present evidence of the complex regulatory network controlling expression of *repA*, *repB*, and *repX* genes and the RA3 copy number; identify the RepA repressor binding sequence in *repAp* and establish the boundaries of *oriV* and the role of transcription in the activation of RA3 *oriV*.

## 2. Results

### 2.1. Wild Type RA3 Minireplicon Encompasses DR1-repA-repB-DR2 and the Junction Region between the Replication and Stability Modules

The dissection of RA3 minireplicon seemed to be straightforward since the in silico analysis of RA3 genome indicated the clustering of elements potentially involved in the replication function (Figure 1) between the integron In10 and the stability module *orf02-orf011* [9]. The three-step procedure was used to create a miniRA3 variant. First, RA3 was digested with SnaBI (one out of three SnaBI sites in RA3 genome was located on the edge of DR2) and self-ligated (Figure S3A). Since the 9 kb SnaBI fragment encompassing DR1-*repA-repB*-DR2 also carried a part of In10, e.g., *aad2* gene, the DH5α transformants might have been selected on media with streptomycin. Second, the Km^R^ cassette from pKRP11 [16] was cloned into the SnaBI site and Sm^R^ Km^R^ DH5α transformants were selected. Third, plasmid DNA was treated with PvuII and self-ligated to remove In10. Kn^R^ Sm^S^ transformants carried the minireplicon consisting of the 3.05 kb PvuII-SnaBI RA3 fragment (Figure 1B, coordinates 44942-2082 nt on the RA3 circular map DQ401103) and the 1.3 kb Kn^R^ cassette. DNA sequencing of the miniRA3-1 (pMOB1.3.2) revealed a point mutation (transversion A→C at position 45534 nt according to the RA3 sequence) leading to the substitution of Thr at position 32 into Pro in the putative H-T-H motif of the RepA sequence (Figure 2A). Since mutation appeared in the SnaBI fragment during the first step of cloning, sixteen other transformants that formed a normal size colony and demonstrated the high plasmid DNA yield and two that grew as very small colonies and gave the low DNA yield, were chosen (Figure S3B) for further analysis.

Sixteen well-grown SnaBI clones carried plasmids with mutations (Figure 2A,B) either in the *repA* gene (13 derivatives) or in the intergenic region *repA-repB* (3 derivatives). The Ala29 in the putative H-T-H motif of RepA seemed to be the most frequently modified residue, into either Val or Gly. Another mutant in the putative H-T-H motif carried T30P substitution as observed for miniRA3-1. The next two mutants had substituted residues in the C-terminus of RepA: L57Q and D76V. The latter variant also had altered D23Q in putative RepX polypeptide. One substitution P9L localized in the N-terminus of RepA. We also found two *repA* mutants with major defects: one introduced a stop codon after residue S4, whereas a single nucleotide deletion caused a simultaneous frame-shift mutation after residue A74 in RepA and P24 in RepX.

Three mutations that arose in the *repA-repB* intergenic region during miniRA3 construction seemed to target predicted *repXp* (Figure 2B). Duplication of the seven nucleotides extended the distance between a putative−35 (TTGCCA) and−10 (TATAAT) motifs from 16 to 23 nt (*repXp-4*). The mutant designated *repXp-1* carried the transition of T→C (*C*ATAAT) in a putative -10 motif. The SnaBI derivative carrying this mutation was used to construct miniRA3-5. The third variant (*repXp-3*) carried transversion T→G in the putative −35 motif for *repXp* (*G*TGCCA).

The attempts to get a good yield of plasmid DNA for sequencing from the clones forming tiny colonies in the initial screening were not successful. Hence, DNA sequencing of the PCR-amplified *repA-repB* regions was performed, which revealed the WT sequence. Altogether, it suggested that SnaBI digestion was removing some important element(s) for the plasmid replication and those mutations either in *repA* or *repXp* restored the ability of replicon to be fully established. This hypothesis proved to be correct when the WT miniRA3 (pJSB18) containing the region between coordinates 43327–45909 and 1–2300 nt was finally obtained. It arose due to the large spontaneous deletion when an attempt to replace the integron sequences in RA3 by Tc^R^ cassette from pKRP12 was undertaken [16] (Figure 2C). The 218 bp fragment downstream of the SnaBI site, at the edge of DR2, that was absent in the mutant variants of the miniRA3 isolated initially (Figure 2C), seemed to be vital for maintaining integrity of the WT RA3 minireplicon sequence. Any attempt to remove the SnaBI-ClaI restriction fragment from pJSB18 led to genetic rearrangements in the 3′ end of *repB* and the DR2 part. The 218 bp region contains divergent face-to-face promoters *orf02p*/ *orf02p*_rev_ [17], where *orf02p* drives expression of the stability module [11] and *orf02p*_rev_ is directed toward DR2 in the replication module. Both promoters are repressed by two global regulators, KorC [17] and KorB [18] repress both promoters. No ORFs could be identified as being expressed from *orf02*p_rev_ transcripts. The analysis of mRNA from *orf02p*_rev_ is discussed in the next sections.

### 2.2. Search for the Plasmid Components Vital for the Minireplicon Function by Use of the Incompatibility Test

To define elements that play important roles in the process of plasmid replication (origin of replication *oriV*, replication factors) or copy number control, the incompatibility test was conducted. The rationale behind such a test is that the presence in the same bacterial cell of these crucial elements, on a high copy number plasmid *in trans* to the minireplicon, disturbs proper functioning of the minireplicon.

**Figure 3:**
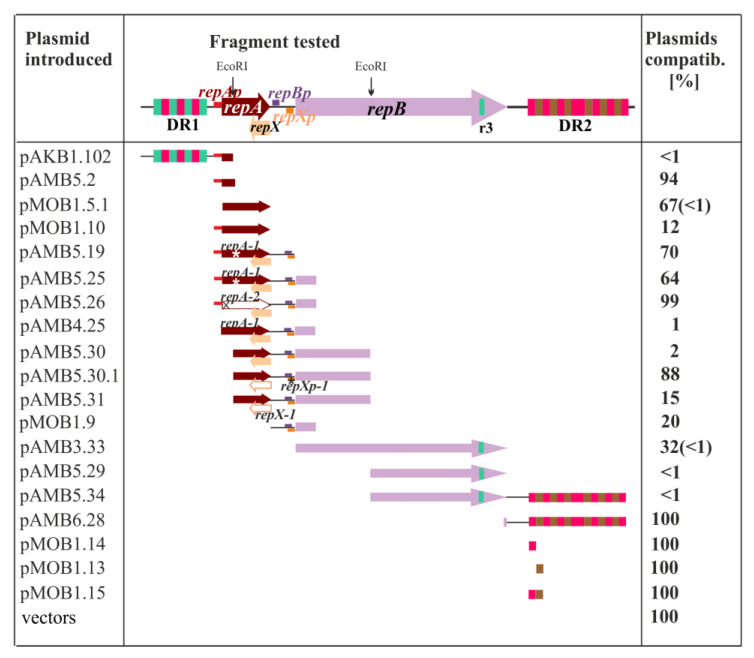
Incompatibility test. The indicated fragments were cloned into high copy number plasmids: pUC18 or pGBT30 and introduced into *E. coli* DH5α (miniRA3-1) strain. The transformants were selected either on Pen plates (incoming plasmid) or Pen and Kan plates (both resident and incoming plasmids). Plasmid compatibility is expressed as the percentage of cells carrying both plasmids versus the number of transformants after selection for the incoming plasmid. In the case of pMOB1.5.1 (*tacp-repA*) and pAMB3.33 (*tacp-repB*), the transformants were selected without or with 0.5 mM IPTG, the *tacp* inducer. The values in the brackets correspond to the level of the co-existence of both plasmids under conditions of RepA or RepB protein overproduction.

The library of different fragments from miniRA3 was constructed by cloning them into the high copy number plasmids, mainly pUC18 but also into pGBT30 (the expression vector based on pMB1) [19]. After plasmids construction, their verification by DNA sequencing and confirmation of the ability of purified plasmid DNAs to efficiently transform *E. coli* DH5α cells, the incompatibility tests were performed (Figure 3).

The DH5α (miniRA3-1) strain was then transformed with a set of the high copy number plasmid derivatives, and transformation mixtures were adequately diluted and spread on plates with penicillin (selection for the incoming plasmid) and on plates with penicillin and kanamycin (selection for both, resident miniRA3-1 and the incoming plasmids). The number of colonies grown on the double selection plates versus the number of colonies grown on the penicillin plates (%) reflected the compatibility of both plasmids, ranging from 100% (fully compatible) to less than 1% (highly incompatible, unable to co-exist in the same cell). Both ORFs, *repA* and *repB*, when cloned into the expression vector pGBT30 under control of *tacp* [19] (pMOB1.5.1 and pAMB3.33) and then introduced to *E. coli* DH5α (miniRA3-1) exerted strong incompatibility when double transformants were grown on IPTG. It suggested the negative regulatory roles in plasmid establishment of both RepA and RepB, when in excess.

The strongest incompatibility (less than 1% of the transformants hosting both plasmids) was observed between miniRA3-1 and the plasmids carrying three different RA3 regions i/ pAKB1.102 with a fragment encompassing DR1 (r1r3 repetitions) and the *repAp* region; ii/ pAMB5.29 and pAMB5.34, two constructs containing the 3′ part of *repB* with a single copy of the partial r3 sequence; and iii/ pAMB5.30 carrying 960 bp EcoRI fragment with the 3′ part of *repA*, the intergenic *repA-repB* region, and the 5′ half of *repB.*

The incompatibility of pAKB1.102 was due to the presence of DR1 since the *repA* promoter region was fully compatible with miniRA3-1 when present on the high copy number plasmid (pAMB5.2). DR1, the 354 bp region upstream of *repA*, is composed of three r1 (38 bp) and four r3 sequences (60 bp). Since no incompatibility was observed between miniRA3-1 and pAMB6.28 carrying DR2 with r1 and r2 sequences, it was concluded that the presence of r3 copies was the main incompatibility determinant in pAKB1.102 as well as in pAMB5.29 and pAMB5.34 (Figure 3), suggesting that r3 may represent a putative iteron, the RepB binding site. Further studies have shown (next section) that a single truncated r3 copy (25 nt) present in the 3′ end of the *repB* is sufficient for the initiation of replication.

Significantly, despite the presence of this partial r3 in pAMB3.33 with the *tacp*-*repB* transcriptional fusion, no such strong incompatibility toward miniRA3-1 was exerted when *tac*p was not induced (30% cells could accommodate both plasmids). This might be explained by the leakiness of *tacp*; thus, together with delivering a RepB binding site, RepB would also be delivered to the cells under these conditions.

The incompatibility exerted by 960 bp EcoRI fragment of RA3 had been reported long time ago [20] and thus recommended for the IncU plasmids typing although neither the sequence nor the function of this DNA fragment had been presented. The structure of this intergenic region and its role in the incompatibility were therefore studied here. The shortening of the EcoRI fragment by removal of a part encoding 5′ end of *repB* (pAMB4. 25) retained strong incompatibility, however, the EcoRI fragment with a mutation in the *repX* promoter (pAMB5.30.1) abolished the strong incompatibility. Finally, a mutation in the start codon of *repX* ORF within the EcoRI fragment partly enhanced the plasmid establishment, 15% clones demonstrated the presence of both tested plasmids. It suggested that the presence of RepX increased the incompatibility caused by the *repA*-antisense transcript coming from *repXp*.

### 2.3. Defining oriV by Deletion and Complementation Analysis

**Figure 4:**
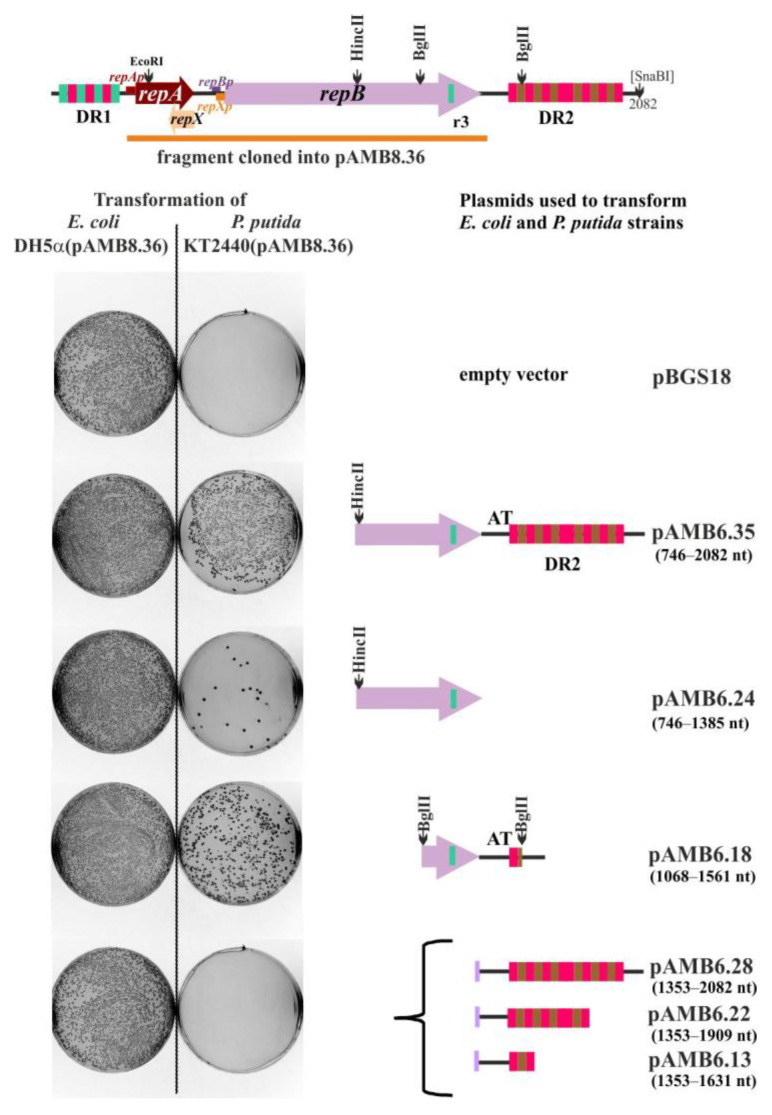
Mapping of the *oriV*_RA3_ region. DNA fragment encoding *repA-1* and *repB* was cloned into the BHR vector (pAMB8.36) to be expressed in various hosts (upper scheme). The miniRA3-1 segments were cloned into pBGS18 as shown on the right. The same DNA quantities of the pBGS18 derivatives were used to transform the competent cells of *E. coli* DH5α (as a control of transformation efficiency) and *P. putida* KT2440 derivatives carrying pAMB8.36. The empty pBGS18 is unable to replicate in *P. putida* strain. The efficiency of the transformation and growth of the double transformants is demonstrated on photographs of the transformation plates selective for both plasmids (left panel).

To define the boundaries of the RA3 *oriV* region, the *repA-repB* coding fragment with the intact upstream regulatory sequences was amplified by PCR from miniRA3-1 template and inserted into the broad-host-range vector pBBR-MCS3 Tc^R^ [20] to obtain a helper pAMB8.36 delivering an initiator replication protein. Various length fragments of the miniRA3 replicon, presumably carrying *oriV*_RA3_, were cloned into pBGS18 and used to transform the *P. putida* strain KT2440(pAMB8.36). The results showed that the RA3 fragment, which initiated replication very efficiently under these conditions, encompassed 3′ end of *repB* genes as well as the downstream region of 556 bp designated DR2 (pAMB6.35) (Figure 4). The presence of insert with the deleted 3′ end of *repB* did not facilitate the pBGS18 derivative replication (pAMB6.28) in *P. putida* KT2440 (pAMB8.36) strain. 

Significantly, the 3′ end of *repB* containing a truncated r3 was scarcely sufficient to support pAMB6.24 replication. The BglII fragment encompassing r3 at the end of *repB* and the AT-rich region upstream of the first r1 sequence in the DR2 (pAMB6.18) elevated the efficiency of replication, however, it was estimated to be at least 10-fold lower than efficiency of the fragment present in pAMB6.35. All tested plasmids were used to transform to the same lot of *P. putida* KT2440(pAMB8.36) competent cells and gave a similar efficiency of control transformation of *E. coli* DH5α. Then, it was concluded that although partial r3 is sufficient for initiation of replication, it is not solely accountable for the full activity of the origin. The AT-rich region and DR2 repeats are required to potentiate the function of *oriV*_RA3_ (Figure S4).

### 2.4. The repA and repB form an Operon and the repXmRNA Is Complementary to repAmRNA over Its Full Length

**Figure 5:**
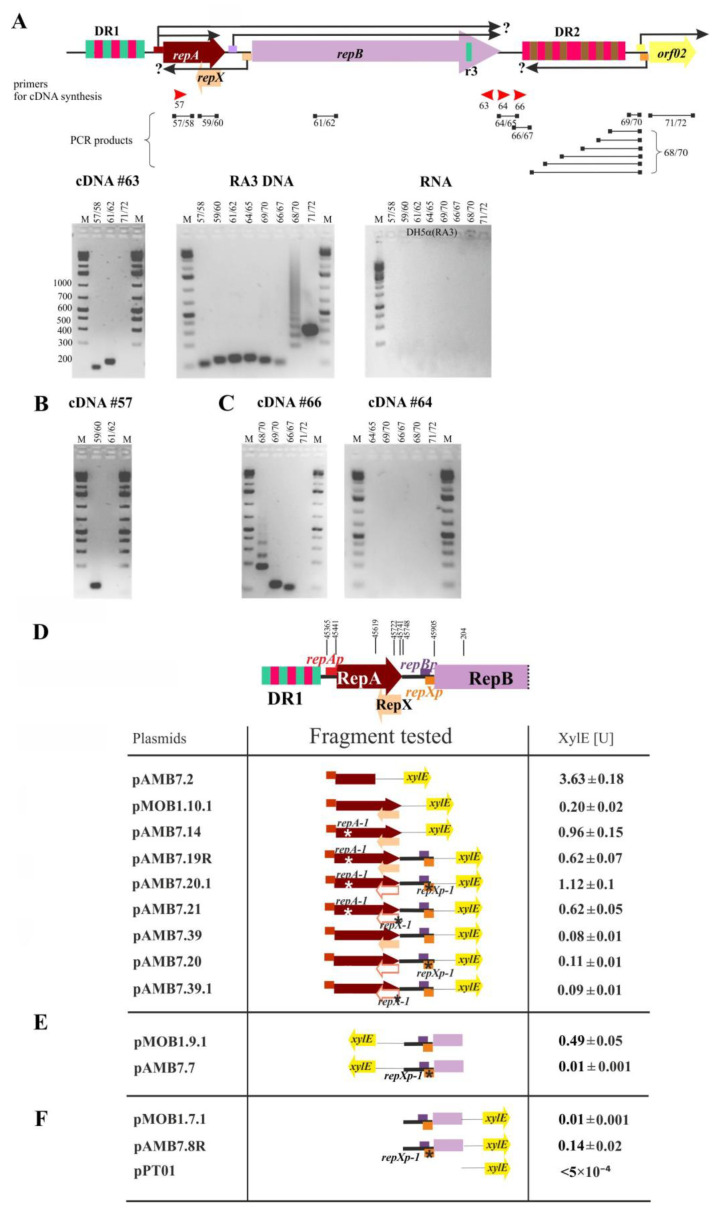
Transcriptional analysis of the *repA-repB* region. (**A**–**C**) RT-PCR analysis of the replication module on RNA isolated from DH5α(RA3). The upper part illustrates the location of primers used for cDNA synthesis (red arrows) and the expected PCR products for various primer pairs. (**A**) RT-PCR analysis of the *repA-repB* co-expression. The results of PCRs on cDNA obtained with primer #63 with indicated pairs of primers are demonstrated in the left photograph. Two control sets of PCRs were conducted on RA3 DNA (middle photograph) and DH5α (RA3) RNA samples. (**B**) RT-PCR analysis of the transcript from *repXp.* cDNA was synthesized on mRNA*repX* with the use of primer #57 and applied in PCRs with the indicated pairs of primers. (**C**) RT-PCR analysis of the extent of the transcript from *orf02p*_rev_. Two primers, #66 (left) and #64 (right) were used for cDNA synthesis. The products of PCRs with the indicated pairs of primers are demonstrated on the gels. M- indicates DNA markers (**D**–**F**) Transcriptional analysis in vivo of the *repA-repB* region by use of the transcriptional fusions. Various fragments of wt or mutant versions of the replication module (depicted on the diagram) were cloned upstream of the promoterless *xylE* cassette in pPT01. The XylE activity was assayed in the extracts from the logarithmic cultures of the appropriate transformants of *E. coli* C600 strain (plasmids listed at the left). (**D**) Analysis of the *repAp-xylE* fusions in the various genetic background. (**E**) Analysis of the *repXp-xylE* fusions. (**F**) Analysis of the *repBp-xylE* fusions. Assays were done at least in triplicate and the results are presented with SD.

Bioinformatic analysis of the RA3 replication module [9] pointed out the three promoter regions in the replication module: *repAp* upstream of *repA* (Figure 1A), and two regions close to the consensus promoters in the intergenic region *repA-repB*: preceding *repB* ORF and oppositely oriented *repXp* (Figure 2B). The present study, additionally, suggests that a fully functional WT minireplicon requires the presence of the *orf02p*_rev_ firing toward DR2. RT-PCR technique was applied to confirm the existence and determine the extent of mRNA from the *orf02p*_rev_ and *repXp*, as well as to verify the hypothesis [9] that *repB* may be expressed not only from the upstream *repBp* but also from the distant *repAp*. The total RNA was isolated from two strains, *E. coli* DH5α (RA3) and DH5α (WT miniRA3). The similar results were obtained for both strains, the results for RNA isolated from *E. coli* DH5α (RA3) strain are presented in Figure 5A–C, the results for RNA isolated from *E. coli* DH5α (WT miniRA3) are included in Figure S5.

First, cDNA was synthesized in the reverse transcriptase reaction on mRNA with the use of the primer annealing to the end of *repB* (#63). Amplification of the *repA* (primers #57/58) on such a cDNA confirmed the existence of the contiguous *repA-repB* transcript (Figure 5A left photograph). Control reaction amplifying *orf02* on the same cDNA gave no product (primers #71/72). The purity of RNA (no DNA contaminations) was verified in PCR reactions with primers used in the further experiments (Figure 5A right photograph). The used primers were verified in PCR on RA3 DNA template (Figure 5A middle photograph).

To estimate the extent of *repX*mRNA, cDNA was synthesized on the same RNA samples with the primer #57 corresponding to the 5′ end of *repA* ORF (Figure 5B). The PCR product obtained with a pair of primers #59/#60 confirmed the production of antisense mRNA overlapping *repA* ORF. The estimated length of *repX*mRNA by northern analysis coincided with this data (see below).

### 2.5. The Extent of mRNA Synthesized from orf02p_rev_ toward oriV

To estimate the boundaries of *orf02p_rev_*mRNA, cDNAs were synthesized in the reverse transcriptase reaction on the RNA isolated from DH5α(RA3) and two primers, either #64 (corresponding to the DNA sequence after the 3′ end of the *repB*) or #66 (corresponding to the region after the AT-rich sequence in the *oriV*) (Figure S4). The RT-PCR products were used as the templates in PCR with various pairs of primers (Figure 5C). The expected products were obtained only on cDNAs synthesized from the primer #66 (Figure 5C left photograph) and were not observed in the reaction when the primer #64 was used for cDNA synthesis (Figure 5C, right photograph). It indicates that mRNA from *orf02p*_rev_ reaches the AT region up to the coordinates 1449 nt but does not extend to the end of *repB* (RA3 coordinates 1366 nt).

### 2.6. Transcriptional Analysis of the repA, repB, and repX Promoters

The 256 bp fragment encompassing putative *repAp* or the 376 bp fragment containing *repAp* followed by the intact *repA* ORF (*repAp-repA*) were fused to the promoter-less *xylE* cassette in pPT01 [21]), giving pAMB7.2 and pMOB1.10.1, respectively. The XylE activity was very high when fusion was expressed from pAMB7.2 (3.63 U) and almost 20-fold lower (0.2 U) in the presence of RepA (pMOB1.10.1). Replacement of WT *repA* in pMOB1.10.1 by the *repA-1* allele (pAMB7.14) elevated the XylE activity up to 0.96 U (Figure 5D). Only a 5-fold increase in the transcriptional activity of the fragment carrying the point *repA-1* mutation in comparison to the wild type sequence suggested that the repressor activity of RepAT32P was impaired but not completely abolished. Altogether it has been concluded that *repAp* is a very potent promoter, strongly autoregulated by WT RepA.

The moderate level of expression (0.49 U) was observed for *repXp* (pMOB1.9.1) with the inserted fragment of 365 bp (RA3 coordinates 186–205 nt). Fragment of the same length but carrying mutations *repXp-1* (T→C substitution in the -10 motif of *repXp,* Figure 2B) showed the activity of *repXp* decreased more than 100-fold (pAMB7.7, Figure 5E).

The low transcriptional activity of *repBp* (0.01U; pMOB1.7.1) coming from the intergenic region *repA-repB* was demonstrated previously [9]. To check whether the stronger divergently oriented *repXp* interferes with the transcription from *repBp* (Figure 2B) the effect of *repXp-1* mutation on the activity of *repBp* was analyzed (pAMB7.8R). The activity of *repBp* in this construct increased 14-fold in comparison to pMOB1.7.1 (Figure 5F), which confirmed the negative interference between *repBp* and *repXp*. The roles of *repXp* and the RepX polypeptide were then analyzed in the context of both *repAp* and *repBp* activities (see below).

When the *repAp-repA-1* region extended by the intergenic *repA-repB* sequence was fused to *xylE* (pAMB7.19R), it demonstrated a decrease of approximately 30% in the transcriptional activity comparing to pAMB7.14 (*repAp-repA-1*). It implicated that only a part of transcripts originated at *repAp* went through the intergenic region into *repB* (Figure 5D). Then, the fragments of the same lengths as those cloned in pAMB7.19R and pAMB7.14 but with WT *repA* allele were analyzed in the *xylE* transcriptional fusions, pAMB7.39 and pOMB1.10.1, respectively. The XylE activities were much lower (as expected for RepA-regulated transcription) but the 30% decrease in the transcriptional activity was also observed between this pair of constructs, which confirmed the partial reading through into the *repB* (Figure 5D).

The presence of *repXp-1* mutation in plasmids pAMB7.20.1 (compared to pAMB7.19R) or pAMB7.20 (compared to pAMB7.39) elevated the transcription from *repAp* and/or *repBp* into *repB* independently of the *repA* status. No effect of the *repX-1* mutation (lack of RepX polypetide synthesis but intact production of *repX*mRNA) was observed on the read-through transcripts (compare XylE activities between pAMB7.39 and pAMB7.39.1 and the second pair pAMB7.19R and pAMB7.21) (Figure 5D). It suggests that transcription from *repXp* by itself and not the product of translation, RepX, plays an important role in controlling the *repB* expression.

### 2.7. Properties of RepA and Its Variants

**Figure 6:**
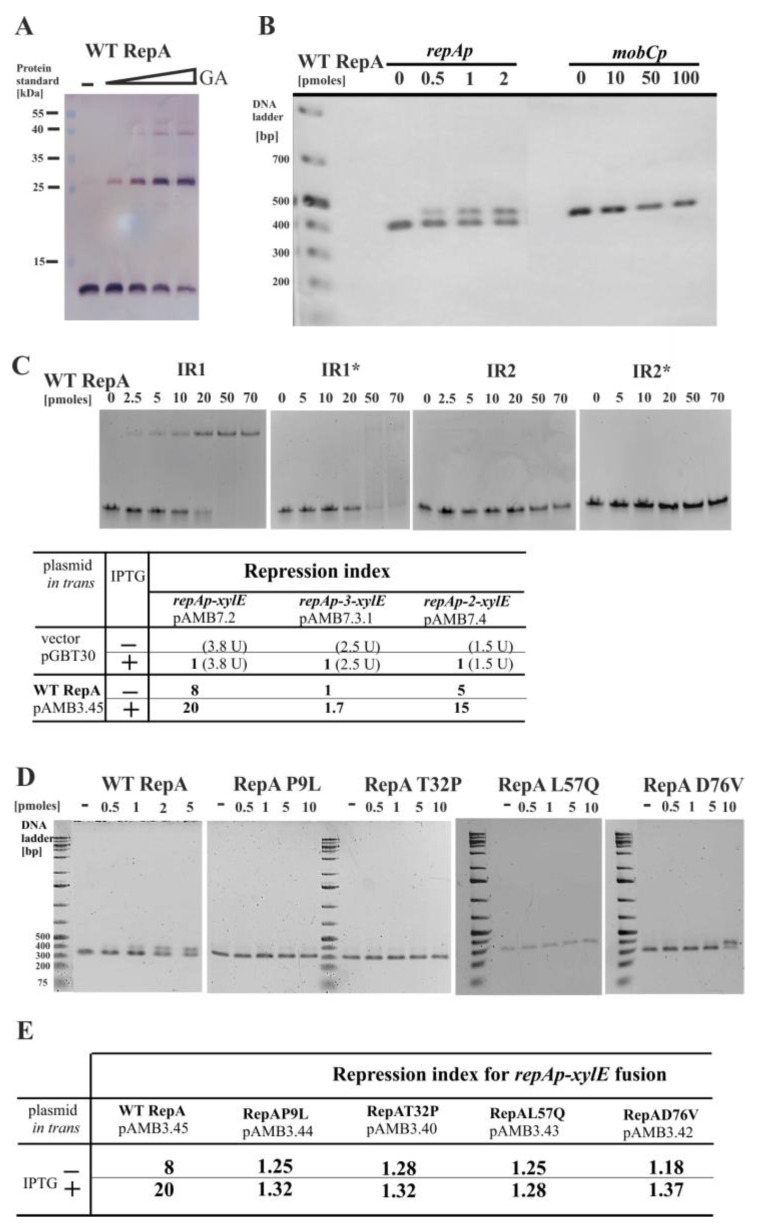
Properties of RepA (**A**) Dimerization in vitro. The His_6_-RepA was overproduced in the cultures of BL21(pAMB11.47) and purified by the affinity chromatography. The purified His_6_-RepA protein (0.025 mg mL^−1^) was incubated with increased concentration (0.001, 0.005, 0.01, and 0.05%) of the cross-linker glutaraldehyde (GA) and complexes resolved by PAGE on 16.5% denaturing polyacrylamide gel. The complexes were transferred to nitrocellulose membrane and visualized by western hybridization with anti-His tag antibodies. (**B**) Specificity of RepA DNA binding activity. The His_6_-RepA protein was incubated with 30 ng of two PCR products corresponding to *repAp* (primers #1 and #2) or *mobCp* (#43 and #44). The complexes were separated at 0.8% agarose gel in 0.5xTBE buffer, ethidium bromide stained, and photographed. (**C**) Identification of the RepA operator. Upper panel: His_6_-RepA protein was incubated with 150 ng of ds oligonucleotides corresponding to the inverted repeats detected in the *repAp* region, IR1 (#45/#46), and IR2 (#47/#48). In parallel, the same length fragments with scrambled inverted repeats were used, IR1* (#49/#50) and IR2* (#51/#52). The products of reactions were separated by PAGE on 10% acrylamide gel in the 0.5xTBE buffer, ethidium bromide stained, and photographed. Bottom panel: The in vivo effect of IR1* and IR2* substitutions in the *repAp-3*-*xylE* and *repAp-2-xylE* transcriptional fusions, respectively. The expression vector pGBT30 and its derivative pAMB3.45 with *repA* ORF cloned under *tacp* were used to transform *E. coli* C600 strain. The 256 bp *repAp*, the *repAp-3*, and *repAp-2* fragments were cloned upstream of the *xylE* cassette in pAMB7.2, pAMB7.3.1, and pAMB7.4, respectively, and used to transform both strains. The XylE activity was assayed in extracts of double transformants grown with and without inducer, 0.5 mM IPTG. Detected XylE activities (U) in the control strains are shown in brackets. Repression index (RI) was calculated as the ratio of the XylE activity detected in the appropriate control strain grown with IPTG and XylE activity in the presence of overproduced RepA. The assays were repeated at least three times. (**D**) DNA binding activities of WT RepA and its mutant variants. Four His_6_-tagged RepA variants with WT His_6_-RepA as a control were used in EMSA with 30 ng of PCR fragment corresponding to *repAp* (#1 and #7). The products of DNA binding reactions were separated on 0.8% agarose gels as in panel B. (**E**) Repression ability of WT RepA and its variants. All mutated *repA* alleles were cloned under *tacp* in pGBT30. XylE activity was assayed in the extracts of double transformants of the C600 strain grown with and without inducer 0.5 mM IPTG. C600(pAMB7.2)(pGBT30) was used as a control. The repression index (RI) was calculated as described in (**C**).

The WT RepA (98 amino acids), His_6_-tagged at the N-terminus was overproduced and purified. Cross-linking experiment with glutaraldehyde revealed the ability to form mainly dimers (Figure 6A). The purified RepA protein was used in the EMSA with a 376 bp PCR amplified *repAp-repA* fragment (RA3 coordinates 45365–45741). The 417 bp PCR product with *mobCp*_RA3_ from the conjugative transfer module (RA3 coordinates 9435–9852) was used as the control of RepA DNA binding specificity. RepA specific binding to the *repAp-repA* fragment was detected at 25 nM, whereas no binding to the control fragment was observed even at 5 µM RepA (Figure 6B).

Sequence analysis of the *repAp* region revealed putative −35 (TTGTTG) and −10 (TACACT) motifs separated by 17 nt. It also showed two imperfect palindromic sequences in this region, designated IR1 and IR2 (Figure S6). The IR1, TTGAAGTtTAcCaactagGtTAcACTTCAA (capital letters indicating complementary bases in the palindromic arms), seems to overlap both *repA* promoter motifs (in bold at Figure S6), whereas IR2 (CaCATCAttcTGATGaG) presumably overlaps a putative transcription start point and rbs for *repA* (underlined at Figure S6). Several nucleotide substitutions into IR1* (tTgaAGTtcgcgaactagcttacACTctAg) or IR2* (CagtcgActcTgatgaG) were introduced to destroy the arms complementarity but trying to avoid the interference with the promoter motifs (Figure S6). The ds oligonucleotides corresponding to IR1, IR2, and their mutated versions IR1* and IR2*, respectively, were used in the EMSA (Figure 6C upper panel). IR1 was bound by RepA at 0.1 µM, IR1* was also bound but with a lower affinity. No RepA binding was detected to IR2 or IR2* even at 4 µM RepA. These results strongly suggested that IR1 represents the RepA operator (O_RepA_) in the *repAp*. The role of IR2 remains unknown.

The same nucleotide substitutions as in IR1* and IR2* were introduced by site-directed mutagenesis into the 256 bp *repAp* fragment to get *repAp-3* and *repAp-2* alleles, respectively. Then, the fragments were cloned into the promoter-probe vector pPT01 [21] to obtain pAMB7.3.1 and pAMB7.4, respectively (Figure 6C bottom panel). The *E. coli* C600 (pAMB7.2 *repAp-xylE*), C600 (pAMB7.3.1 *repAp-3-xylE*), and C600 (pAMB7.4 *repAp-2-xylE*) strains were transformed either with the empty expression vector pGBT30 [19] or its derivative pAMB3.45 (*tacp-repA*). The cultures were grown with and without IPTG inducer and XylE activity was assayed in the cell extracts. The levels of *repAp* and *repAp-2* (IR2*) expression were strongly reduced in the presence of RepA (Repression Index RI was 8 even without *tacp* induction and up to 20 after induction). The expression of *repAp-3* (IR1*) was hardly responding to the presence of RepA even when overproduced (RI in the range of 1 to 1.7).

In the next step, four variants of RepA, the products of the mutated alleles obtained spontaneously during minireplicon cloning (Figure 2A), were tested for the ability to repress *repAp* and to bind in vitro to the purified 256 bp PCR fragment carrying *repAp*. In the EMSA with the His_6_-tagged purified RepA variants, three of the derivatives, RepAP9L, RepAT32P, and RepAL57Q showed no DNA binding under tested conditions, whereas RepAD76V exhibited 5-fold lower affinity toward *repAp* (Figure 6D upper panel). None of the four derivatives repressed *repAp-xylE* fusion in the in vivo regulatory test giving RI around 1 (Figure 6D bottom panel). Hence, it was confirmed that spontaneous mutations arisen during minireplicon isolation had significantly impaired the autoregulatory activity of RepA.

### 2.8. The Copy Number of miniRA3 Depends on repAp, repBp, and repXp Transcription and Presence of RepX

The plasmid copy number control is usually exerted by the level of synthesis of the replication initiator, its activity, and its *oriV* accessibility. The copy numbers of RA3, WT miniRA3 (pJSB18), and five minireplicon variants were estimated in *E. coli* DH5α strain by the use of Real-time qPCR and pairs of primers amplifying a *repB* fragment versus a fragment of chromosomal *galK*. The copy number of RA3 and WT miniRA3 was estimated as one copy per chromosome (Table 1), so it confirmed that the copy number control circuits remained intact in the WT miniRA3. Significantly, the copy number of miniRA3-1 carrying the spontaneous mutation in the *repA* gene (*repA-1*), although still very low, was elevated to 2–3 copies per chromosome. It suggested the participation of either RepA or the 218 bp region downstream of DR2 in controlling the copy number of the intact RA3 plasmid.

The mutant miniRA3-2, derivative of miniRA3-1 with additionally impaired RepA operator O_repA_ (IR1*), demonstrated a very high copy-number of 75 copies per chromosome. Another derivative of miniRA3-1, the miniRA3-7, with modified IR2 in the *repAp* had an increased copy number to 43 copies per chromosome. The lack of RepX synthesis in miniRA3-4 significantly increased the copy number of the minireplicon in comparison to the parental miniRA3-1, i.e., to 14 copies per chromosome. Significantly, the *repXp-1* mutation in the miniRA3-5 had the same effect on the minireplicon copy number as the *repA-1* mutation (Table 1).

Stability of RA3, WT miniRA3 (pJSB18), and five mutated minireplicons was analyzed in the *E. coli* DH5α strain during growth without selection. As expected, RA3 was very stably maintained (100% retention) for at least 60 generations (Table 1). Surprisingly, WT and the mutant miniRA3 derivatives were also very efficiently retained despite the removal of the *orf2-orf11* region encoding active partition operon [11,22].

The copy number and stability of the RA3 derivatives were also analyzed in *P. putida* KT2440 cells (Table 1). RA3 and WT miniRA3 exhibited the same copy number in *P. putida* as they did in *E. coli*. Analyzed mutations in minireplicons led to the increase in the copy number up to 10 per chromosome and the significant increase in the stability of the mutated variants. The effects of the mutations on the plasmid copy number in the *P. putida* host did not correspond to that observed in *E. coli.*, e.g., no effect of the RepX absence was observed in KT2440 strain. It suggests that mechanisms controlling the plasmid copy number may be species-specific.

The RA3 was quite unstable in *P. putida* since after 60 generations of growth in nonselective medium it was only retained in 10% of cells and WT miniRA3 was extremely unstable being lost by 97% of cells during 20 generations of growth without selection. MiniRA3-1(*repA-1*) and miniRA3-4 (*repA-1, repX-1*) demonstrated the highest retention being present in 30% of cells after 60 generations.

### 2.9. Northern Analysis of the Various Transcripts Produced by the Minireplicon

**Figure 7:**
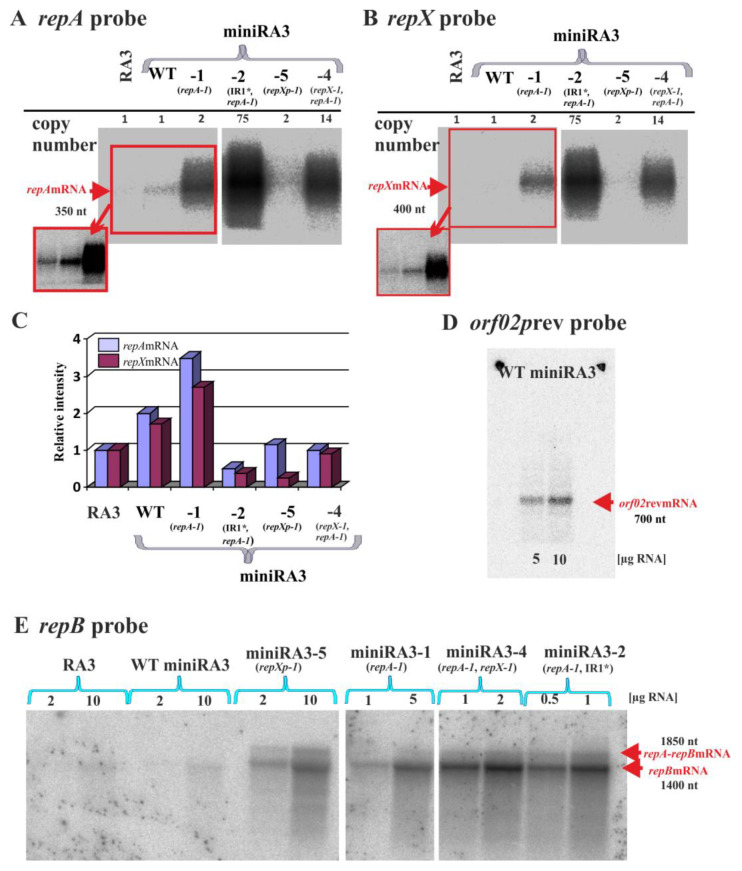
Northern analysis of the transcripts in the RA3 replication module. 0.5–12 μg of total RNA isolated from the transformants of DH5α strain with RA3, WT miniRA3, or four miniRA3 mutants was denatured and separated on the denaturing agarose gels. The RNA from the gels was transferred onto a nitrocellulose membrane that was hybridized with four different radioactive probes. (**A**) and (**B**) DNA-RNA hybridization to visualize *repA*mRNA and *repX*mRNA, respectively. Of the total RNA, 12 µg was used in experiments. Mutations present in the minireplicons are indicated. The copy number of the analyzed plasmids is shown above the autoradiographs. The parts of gels in the insets demonstrate the longer exposures. (**C**) The diagram demonstrates the relative intensity of signals from gels (**A**,**B**) in comparison to RA3 mRNAs. Photostimulated luminescence (PSL/mm^2^) detected for *repA*mRNA and *repX*mRNA was normalized to the single plasmid copy. (**D**) Visualization of *orf02*_rev_mRNA produced from WT miniRA3 by DNA–RNA hybridization. (**E**) Visualization of the *repB* transcripts in RNA–RNA hybridization. Various amounts of RNA were tested as indicated for each track.

Northern analysis was applied to directly visualize various transcripts produced in the replication module. RNA was isolated from *E. coli* DH5α carrying RA3, WT miniRA3 and four chosen derivatives, miniRA3-1, miniRA3-2, miniRA3-4, and miniRA3-5, respectively. Initially, three different 5′ end labeled radioactive probes, complementary to *repA*mRNA, to *repX*mRNA, and to the putative transcripts originated at *orf02p*_rev_, were used for DNA-RNA hybridization. Based on the RNA standards applied during the analysis, the *repA*mRNA had an estimated length of 350 nt, whereas *repX*mRNA had 400 nt, respectively. The very low signals were obtained for both these mRNAs in RA3 and WT miniRA3 (Figure 7A,B). The higher levels of both mRNAs were detected in all tested mutant minireplicons except miniRA3-5 (*repXp-1*). The intensities of signals varied between derivatives as expected for replicons of variable copy number, however it was not directly proportional to the estimated plasmid copy numbers, e.g., for miniRA3-1 and miniRA3-2 (see plots below Figure 7C).

With the use of a radioactive probe complementary to mRNA initiated at *orf02p*_rev_, a transcript of approximately 700 nt was detected in RNA samples from the WT miniRA3 (Figure 7D) but not RA3. The inability to visualize the *orf02p*_rev_ transcript from RA3 template may reflect the tight regulation of this promoter by KorC [17]. The *orf02*_rev_ promoter was mapped between coordinates 2200 nt and 2236 nt [17]. The length of this transcript estimated by Northern technique coincided with RT-PCR data (Figure 5C) that pointed out its end close to the 5′ end of the repetitive region DR2 at 1512 nt. Altogether, we concluded that transcripts from *orf02p*_rev_ terminated in the proximity of *oriV*_RA3_ region.

Significantly, when the *repA* probe was applied to visualize the miniRA3 transcripts by DNA-RNA hybridization no predicted *repArepB*mRNA was detected (Figure 7A). It was assumed to result from the inefficient transfer either of the longer size mRNAs or less numerous *repA-repB* transcripts undetectable with the use of this probe. In the second approach, the blotting method was adapted to the large RNA fragments and a highly radioactive RNA probe complementary to *repB*mRNA was used for RNA–RNA hybridization (Figure 7E). In these experiments two transcripts of approximately 1500 and 2000 nt were detected, as expected for *repB*mRNA and *repArepB*mRNA, respectively, with *repB*mRNA being produced in higher quantities.

The most intensive radioactive signals related to the 1 µg of RNA loaded on the gel were detected for miniRA3-4 (*repA-1*, *repX-1*) and miniRA3-2 (*repA-1* IR1*) with the highest plasmid copy number of 14 and 75, respectively. The lower signals were noticed for miniRA3-5 (*repXp-1*) and miniRA3-1 (*repA-1*), whereas the lowest signals were detected for RA3 and WT miniRA3 (Figure 7C).

## 3. Discussion

IncU plasmids are widely spread in the aquatic environment [23]. They are carriers of a variety of antibiotic resistance markers [5,6,23,24]. The ability of conjugative transfer to species from *Alpha*-, *Beta*- and *Gammaproteobacteria* combined with the replication and the long-term maintenance in various hosts make them the significant players in bacterial adaptation and evolution. The RA3 plasmid, as the archetype of the IncU group, has already been thoroughly studied with respect to its stability, expression of the conjugative transfer module, and existence of the regulatory network [9,10,11,17,18,22,25,26,27]. The organization and functioning of the replication module are presented in this article.

The replication module consists of three ORFs, encoding an initiator protein RepB, a regulatory protein RepA and an accessory polypeptide RepX (Figure 1B). The *repA* and *repB* form an operon transcribed from a strong *repAp*, autoregulated by RepA. In this operon, there is also a seemingly much weaker internal *repBp* (Figure 5) oriented face-to-face with the divergent stronger *repXp*. Studies on the separate promoter regions or their combinations cloned in the promoter-probe vector revealed strong repression of *repAp* by RepA and reading -through from *repAp* toward *repB*. Active *repXp* potentially switches off *repBp* as shown after cloning the WT and mutated regions in the promoter probe vector. However, northern analysis of the transcripts’ levels, when all elements of this regulatory puzzle are present, suggests that transcription from *repXp* (RNAP complex, newly synthesized transcript, or its product RepX polypeptide) regulates the balance between *repArepB*mRNA and *repB*mRNA in the advantage of the latter.

RepA strongly represses the expression of *repAp* by binding to IR1 (O*_repA_*), which overlaps the -35 and -10 motifs (Figure 6B,C, Figure S2). Variants of RepA with amino acids substitutions in the predicted centrally located H-T-H motif significantly diminished DNA binding activity of RepA in vitro and in vivo (Figure 6C), increasing the production of not only the long *repArepB*mRNA but also the *repB* transcripts (Figure 5D and Figure 7E). Variants of RepA with amino acid substitutions in the N-terminus (RepA P9L) or C-terminus (RepA L57Q and RepA D76V) were also impaired in DNA binding. Since modified residues are conserved in homologs of RepA (Figure 2A) they may play an important role in the protein oligomerization or proper folding. Importantly these *repA* mutations had arisen during the minireplicon construction as compensation against the removal of the 200 bp fragment seemingly outside of the replication module (Figure 2C). This fragment contains two strong divergent promoters, the *orf02p*_rev_ firing toward the DR2 region and the face-to-face oriented *orf02p*, driving transcription of the stability module [11]. Both promoters *orf02p* and *orf02p*_rev_ are tightly regulated by two RA3 global regulators, KorC [17] and KorB [18]. The northern studies confirmed the production of approximately 700 nt *orf02*_rev_mRNA and the end of this mRNA was mapped just upstream of DR2 sequence close to the AT-rich sequence by RT-PCR (Figure 5C). Since the deletion analysis mapped the minimal *oriV* into the partial r3 motif in the end of *repB* and adjacent AT-rich sequence (Figure 4) we hypothesize that transcription coming from *orf02p*_rev_ activates the origin allowing the RepB protein and/or replisome to facilitate the initiation of replication. Thus, the subsequent rounds of replication would be indirectly under control of two global repressors, KorC and KorB. It could clearly explain the observations that RA3 was quickly lost in *E. coli* strain overproducing either KorC [17] or KorB [18]. Without the transcriptional activation, the higher level of initiator RepB is required and this is achieved in the truncated minireplicons by increasing the read-through transcription from *repAp* into *repB* (*repA* mutants) or increasing the transcription from the internal *repBp*.

One class of the compensatory mutations arisen in the truncated minireplicons mapped in the *repXp* (Figure 2B) suggesting the important regulatory role of either the transcript or its product RepX in the expression of *repB*. No regulatory effect on the initiation of transcription has been assigned to RepX [9]. Thus, it implicated that the face-to-face orientation of a weak *repBp* and a stronger *repXp* had the negative impact of the latter over *repBp* expression. The interplay between both promoters was confirmed by the transcriptional fusions study. The presence of *repXp-1* mutations in the tested *repBp/repXp* fragment 50-fold diminished the activity of *repXp* (Figure 5E, pMOB1.9.1, and pAMB7.7) and 14-fold increased *repBp* activity in the opposite orientation (Figure 5F, pAMB7.8R versus pMOB1.7.1). The northern analysis was in agreement with this result, showing significant overproduction of *repB*mRNA in the miniRA3-5 (*repXp-1*) in comparison to the WT minireplicon (Figure 7E).

The visualization of the transcripts demonstrated that in RA3 and WT miniRA3 all four main transcripts: *repA*mRNA, antisense *repX*mRNA, *repB*mRNA, and readthrough *repArepB*mRNA were produced at a very low steady-state level giving one plasmid copy per chromosome. Accordingly the mutations *repA-1*, *repXp-1*, *repX-1*, IR1*, and IR2* clearly changed the mRNAs levels and this way led to the variability in the plasmid copy numbers (Table 1). Interestingly, the northern analysis of the mutant versions of minireplicon ended in two major conclusions: i/ the proportion between *repA*mRNA and antisense *repX*mRNA was somehow retained despite the introduced changes and ii/ *repB*mRNA was constantly produced in the higher quantities than *repArepB*mRNA. The latter suggested that reading-through from *repAp* toward *repB* is the back-up mechanism.

The mechanism correlating the levels of the short *repA* transcripts and antisense *repX* transcripts is still unclear. We hypothesize that the important role is being played by Rho-independent divergent transcriptional terminator at the end of *repA* ORF (Figure S2). The terminatory activity seems to be stimulated by transcription from *repXp* leading to a rise of the short *repA*mRNA and the expression of *repB* relying only on the *repBp*. Diminished activity of *repXp* in miniRA3-5 resulted in the lower level of *repX*mRNA in parallel to the lower level of *repA*mRNA and shifted the balance toward reading-through and synthesis of long *repArepB*mRNAs and *repB*mRNA (Figure 7).

So far, we have not completely deciphered the regulatory circuit of *repXp*, the role of the long imperfect IR overlapping *repXp* and RepX itself. Our incompatibility studies confirmed the role of *repX*mRNA in excluding miniRA3 when *in trans.* Since there is an approximately 60 nt overlap between *repX*mRNA (Figure 2B) and complementary *repB*mRNA (Figure S2), it may result in the negative interference diminishing expression of *repBp.* The presence of stop codon in the 5′ end of *repX* ORF significantly relieved the incompatibility effect the transcript played, suggesting an accessory function of RepX in this process.

The RA3 is very stably maintained in some bacterial species, e.g., *E. coli*. Its persistence depends on the active partition system IncC-KorB-*parS* supported by the presence of *kfr* genes, *klcA*, and *orf02* of unknown function [10,11]. The efficient conjugative transfer system may also influence its stability in the population. The most intriguing is, however, the extreme stability of its minireplicon in the *E. coli* host. The removal of the stability and conjugative modules to construct WT miniRA3 did not diminish plasmid retention for 60 generations, despite 1–2 plasmid copies per chromosome. No such stability of miniRA3 is observed in *P. putida* although some analyzed mutations increased the maintenance of the minireplicons. It strongly suggests that on the one hand the regulatory circuits function differently in various hosts, and on the other hand that there is some replication module-encoded factor that stabilizes plasmid specifically in *E. coli*. One of the intriguing hypotheses implicates the role of RepA as an antitoxin HigA homolog that may support the functioning of the innate HigAB system encoded in the chromosome of *E. coli* K12 [28].

The studies on the RA3 replication module revealed the multivalent regulatory network facilitating the plasmid establishment. Despite the clearly modular structure, RA3 replication, stability, and conjugative functions are strongly intertwined. Further studies are required to understand the species-specific functioning of the replication module regulatory network and roles of particular elements.

## 4. Materials and Methods

Bacterial strains and growth conditions

*E. coli* strains used were K-12 strain DH5α F^−^(ɸ80*dlacZΔM15*) *recA1 endA1 gyrA96 thi-1 hsdR17(r_k_^−^m_k_^+^) supE44 relA1 deoR Δ(lacZYA-argF)U19*) [29], C600K (*thr-1 leu-6 thi-1 lacY1 supE44 ton21 galK*) [30], and BL21(DE3) [F^−^*ompT hsdS_B_ (r_B_^−^m_B_^−^) gal dcm* (DE3)] (Novagen, Sigma-Aldrich Pl.). *P. putida* KT2440 strain was kindly provided by C.M. Thomas, University of Birmingham, United Kingdom.

Bacteria were grown in L broth or L agar (L broth with 1.5% *w*/*v* agar) at 37 °C (*E. coli*) or 30 °C (*P. putida*), supplemented with appropriate antibiotics. For *E. coli*, benzyl penicillin, sodium salt was used at 150 µg mL^−1^ in liquid media and 300 µg mL^−1^ in agar plates for penicillin resistance, kanamycin sulphate (50 µg mL^−1^) for kanamycin resistance, streptomycin sulphate (20–30 µg mL^−1^) for streptomycin resistance, tetracycline (10 μg mL^−1^) for tetracycline resistance, and chloramphenicol (10 µg mL^−1^) for chloramphenicol resistance. For *P. putida*, tetracycline (25 µg mL^−1^) and kanamycin sulphate (50 µg mL^−1^) were used. The L agar used for blue/white screening contained IPTG (0.1 mM) and X-gal (40 μg mL^−1^).

Plasmid DNA isolation, analysis, DNA amplification, and manipulation

Plasmid DNA was isolated and manipulated using standard methods [31] or kits using manufacturers’ instructions. All new plasmid constructs were verified by DNA sequencing at the Laboratory of DNA Sequencing and Oligonucleotide Synthesis, Institute of Biochemistry and Biophysics Polish Academy of Science. The list of plasmids used and constructed in this study is presented in Table 2. Standard PCR reactions [32] were performed with RA3 DNA or minireplicon derivatives as the templates and pairs of appropriate primers listed in Table 3.

PCR based site-directed mutagenesis–mutagenesis in situ (Thermo Fisher Sci.PL)

To perform plasmid DNA site-directed mutagenesis, two complementary primers corresponding to the modified region of the template were designed. To simplify the screening of the recombinants, a restriction cleavage site was introduced or inactivated on the newly synthesized primers. PCR was set up in a final volume of 50 μL and the mixture contained 20–100 ng of the template, 1x buffer for Pfu DNA polymerase, 2 nM dNTPs mix, 125 ng of each of the mutagenic primers, and 2.5 U of the high fidelity Pfu DNA polymerase. PCR conditions were as recommended. DpnI endonuclease was added to digest the methylated template. The DpnI treated PCR mixtures were used to transform *E. coli* DH5α strain. Plasmid DNAs isolated from transformants were screened for change in the restriction pattern. Plasmid DNA from a putative mutant was purified and sequenced to verify the introduction of the intended nucleotide substitutions.

Determination of catechol 2,3-oxygenase activity (XylE)

Catechol 2,3-oxygenase activity (the product of *xylE*) was assayed [33] in logarithmically growing bacteria. Plasmid content of all assayed cultures was monitored to ensure that differences in XylE activity were not due to variations in the plasmid copy number. One unit of catechol 2,3-oxygenase is defined as the amount needed to convert 1 μmol of catechol in 1 min under standard conditions. Protein concentration was determined using the Bradford method [34]. Experiments were performed at least in triplicate, and the mean values with standard deviations <10% were reported.

Transformation procedures

*E. coli* and *P. putida* competent cells were prepared by CaCl_2_ treatment [31]. Electroporation was carried out using cuvettes with 2 mm gaps at 25 µF, 200 W, and 2.5 kV in a Bio-Rad Gene Pulser.

Overproduction and purification of His_6_-tagged RepAs by affinity chromatography

For protein overproduction, *E. coli* strain BL21(DE3) carrying pET28mod derivatives, encoding the His_6_-tagged RepA variants was used. The purification procedure was performed as previously described via affinity chromatography [27]. The protein purification was monitored by SDS-PAGE using the PhastSystem (Pharmacia). Protein concentration was determined using the Bradford method [34].

Crosslinking with glutaraldehyde

The standard cross-linking reaction was set in a final volume of 20 μL as described before [35]. The samples were boiled and then separated on 16.5% gels by SDS-PAGE, transferred onto a nitrocellulose membrane, and analyzed by western blotting.

Plasmid copy number

The copy number of plasmids was estimated by real-time qPCR. Transformants were grown on L broth with a selective antibiotic to the stationary phase. Total bacterial DNA was extracted using a modification of the method of Chen and Kuo [36]. Fifty and 100 nanograms of each DNA template were used in qPCRs with 5 µL Hot FIREPol EvaGreen qPCR Mix Plus (Solis Biodyne), and reactions were carried out according to the manufacturer’s instructions in the LightCycler 480 Instrument II (Roche). For RA3 derivatives, the 130 bp fragment from *repB* was amplified with primers #53 and #54, whereas for the chromosomal DNA the same size fragment from *galK* of *E. coli,* primers #55 and #56, were applied. The primers were checked for specificity and efficiency (only primers with an amplification factor between 1.95 and 2 were used). All qPCRs were done in triplicate. The PCN, defined as the number of plasmid amplicons relative to the number of chromosome amplicons, was calculated considering the amplification efficiencies of the primers used [36]. The average results of at least four biological replicates with standard deviation < 10% were analyzed.

**Table 2:** Plasmids used in this work.

Designation	Description	Additional Information
Plasmids provided by others		
pABB21	*ori*_RA3_, Cm^R^, transcriptional terminator T*_tnp513_*from RA3, vector based on the miniRA3-1	[37]
pABB21.1	derivative of pABB21 with truncated DR1 [r3r1] and DR2 [r1r2r1] in the miniRA3-1	A. Bartosik
pABB21.2	derivative of pABB21 with truncated DR1 [(r3r1)_2_] and DR2 [(r1r2)_3_r1(r1r2)r1] in the miniRA3-1	A. Bartosik
pAKB1.102	pGEM_T-Easy derivative, 1174 bp RA3 fragment DR1 *repAp*	A. Kulinska
pBBR1MCS-3	IncA/C, Cm^R^, broad-host-range (BHR) vector	[38]
pBGS18	*ori*_MB1_, Km^R^, high copy	[39]
pET28a	*ori*_MB1_, Km^R^, T7*p*, *lacO*, His_6_-tag, T7 tag, medium copy	Novagen
pGBT30	*ori*_MB1_, Ap^R^, *lacI^q^ tacp*, expression vector, high copy	[19]
pGEM-T-Easy	*ori*_MB1_, Ap^R^, cloning vector, high copy	Promega
pJSB18	**WT miniRA3**, Tc^R^ (RA3 coordinates 1–2300;43327–45909)	J. Godziszewska
pKRP11	*ori*_MB1_, Ap^R^, Km^R^, high copy	[16]
pKRP12	*ori*_MB1_, Ap^R^, Tc^R^, high copy	[16]
pPT01	*ori*_SC101_, Km^R^, medium copy	[21]
pUC18	*ori*_MB1_, Ap^R^, high copy	[40]
RA3	IncU, Cm^R^, Sm^R^, Su^R^, 45.9 kb BHR, conjugative, low copy number plasmid	F. Hayes
**Plasmids constructed during this work**		
	**RA3 derivatives**	
pAMB1.1	9 kb self-replicating SnaBI restriction fragment from RA3, Sm^R^ (RA3 coordinates 1–2082; 38989–45909)	*repA* mutant, (RepAD76V) mutation A→T at position 45667
pAMB1.2	9 kb self-replicating SnaBI restriction fragment from RA3, Sm^R^ (RA3 coordinates as above)	WT
pAMB1.3	9 kb self-replicating SnaBI restriction fragment from RA3, Sm^R^ (RA3 coordinates as above)	*repA* mutant, (RepAD52N), mutation G→A at position 45594 in *repA*; deletion of 1 nt at position 45661 causing frame-shift after 74th codon of RepA and 25th of RepX
pAMB1.4	9 kb self-replicating SnaBI restriction fragment from RA3, Sm^R^ (RA3 coordinates as above)	*repA* mutant, (RepAA29G), mutation C→G at position 45526
pAMB1.5	9 kb self-replicating SnaBI restriction fragment from RA3, Sm^R^ (RA3 coordinates as above)	*repA* mutant, (RepAL57Q), mutation T→A at position 45610
pAMB1.6	9 kb self-replicating SnaBI restriction fragment from RA3, Sm^R^ (RA3 coordinates as above)	*repA* mutant, (RepAP9L), mutation C→T at position 45466
pAMB1.7	9 kb self-replicating SnaBI restriction fragment from RA3, Sm^R^ (RA3 coordinates as above)	*repXp-3* mutant, mutation A→C at position 45890, change in a putative -35 motif for *repXp*
pAMB1.8	9 kb self-replicating SnaBI restriction fragment from RA3, Sm^R^ (RA3 coordinates as above)	*repA* mutant, mutation C→A at position 45451 introducing stop codon in *repA* after 3th codon
pAMB1.9	9 kb self-replicating SnaBI restriction fragment from RA3, Sm^R^ (RA3 coordinates as above)	*repXp-4* mutant, 7 nt duplication between putative -35 and -10 motifs of *repXp* (duplication starts at position 45875)
pAMB1.10	9 kb self-replicating SnaBI restriction fragment from RA3, Sm^R^ (RA3 coordinates as above)	*repA* mutant, (RepAA29V) mutation C→T at position 45526
pAMB1.11	9 kb self-replicating SnaBI restriction fragment from RA3, Sm^R^ (RA3 coordinates as above)	*repA* mutant (RepAT30P), mutation A→C at position 45528
pAMB1.14	9 kb self-replicating SnaBI restriction fragment from RA3, Sm^R^ (RA3 coordinates as above)	*repXp-1*, mutation A→G in a putative -10 motif for *repXp* (position 45869)
pAMB1.14.1	pAMB1.14 derivative, Km^R^ cassette from pKRP11 inserted as the HincII fragment into SnaBI site; Sm^R^, Km^R^	*repXp-1*, mutation A→G in a putative -10 motif for *repXp* (position 45869)
pAMB2.2	**miniRA3-2** *repA-1*, *repAp-1* (IR1*), Km^R^, PCR based- site directed mutagenesis of pMOB1.3.2 (miniRA3-1) with primers #25 and #26	*repA-1* mutation A→C at position 45534; *repAp-1*(IR1*), 3 nt modified in the IR1
pAMB2.3	**miniRA3-7** *repAp-2* (IR2*); Km^R^, PCR based- site directed mutagenesis of pMOB1.3.2 with primers #27 and #28	*repA-*1, *repAp-2* (IR2*), 5 nt modified in the IR2; *repA-1* allele mutation A→C at position 45534
pAMB2.4	**miniRA3-4***, repA-1, repX-1*; Km^R^, PCR based- site directed mutagenesis of pMOB1.3.2 with primers #29 and #30	*repA-1* allele, mutation A→C at position 45534, *repX-1,* mutation A→G at position 45734 eliminating codon ATG for RepX;
pAMB2.7	**miniRA3-5**, *repXp-1*; Km^R^, pAMB1.14.1 digested with PvuII and self-ligated; (RA3 coordinates 1–2082; 38989–39371; 44942–45909)	*repXp-1*, mutation A→G in a putative -10 motif of *repXp* (position 45869)
pAMB2.11	**miniRA3-5**, *repXp-1,* EcoRI*; Km^R^, PCR based-site directed mutagenesis of pAMB2.7 with primers #37 and #38	*repXp-1*–mutation A→G in a putative -10 motif of*repXp* (position 45869); inactivation of EcoRI site within *repA* gene without a change in RepA amino acid sequence;
pMOB1.3	9 kb self-replicating SnaBI restriction fragment from RA3, Sm^R^ (RA3 coordinates 1–2082; 38989–45909)	*repA-1* mutant*,* (RepAT32P), mutation A→C at position 45534
pMOB1.3.1	pMOB1.3 derivative, Km^R^ cassette from pKRP11 inserted as the HincII fragment into SnaBI site, Sm^R^ Km^R^	*repA-1* mutant*,* (RepAT32P), mutation A→C at position 45534
pMOB1.3.2	**miniRA3-1**, *repA-1*; pMOB1.3.1 digested with PvuII and self-ligated, Km^R^; (RA3 coordinates 1–2082; 38989–39371; 44942–45909)	*repA-1* mutant*,* (RepAT32P), mutation A→C at position 45534
pMOB1.16	pMOB1.3.2 derivative; PCR based site-directed mutagenesis with primers #73 and #74 to inactivate EcoRI site in the *repB*	WT *repB,* inactivated EcoRI site within *repB* gene without a change in the amino acid sequence
	**pGBT30 derivatives**	
pAMB3.33	*tacp-repB*; 1385 bp PCR amplified fragment on pMOB1.16 with primers #10 and #16 cloned between EcoRI-SalI sites (RA3 coordinates 1–1385)	
pAMB3.36	*repAp repA-1 repBp* [*repXp repX*] *repB*; 538 bp PCR fragment amplified on miniRA3-1 with primers #1 and #17 cloned as the BamHI-EcoRI fragment into pAMB3.33	*repA-1* allele*;* mutation A→C at position 45534 (RepAT32P); (RA3 coordinates 45365–45903; 1–1385)
pAMB3.40	*tacp-repA-1*; 300 bp PCR fragment amplified on miniRA3-1 with primers #19 and #3 cloned as the SacI-SalI fragment (RA3 coordinates 45441–45741)	*repA-1* allele; mutation A→C at position 45534 (RepAT32P)
pAMB3.42	*tacp-repA-3*; 300 bp PCR fragment amplified on pAMB1.1 with primers #19 and #3 cloned as the SacI-SalI fragment (RA3 coordinates as above)	*repA-3* allele; mutation A→T at position 45667 (RepAD76V)
pAMB3.43	*tacp-repA-4*; 300 bp PCR fragment amplified on pAMB1.5 with primers #19 and #3 cloned as the SacI-SalI fragment (RA3 coordinates as above)	*repA-4* allele*;* mutation T→A at position 45610 (RepAL57Q)
pAMB3.44	*tacp-repA-5*; 300 bp PCR fragment amplified on pAMB1.6 with primers #19 and #3 cloned as the SacI-SalI fragment (RA3 coordinates as above)	*repA-5* allele; mutation C→T at position 45466 (RepAP9L)
pAMB3.45	*tacp-repA*; 300 bp PCR fragment amplified on pAMB2.11 with primers #6 and #3 cloned as the EcoRI-SalI fragment (RA3 coordinates as above)	WT *repA*; inactivated EcoRI site within *repA* gene without a change in the amino acid sequence
	**pGEM-T Easy derivatives**	
pAMB4.25	*repA-1 repBp* [*repXp repX*]; 684 bp PCR fragment amplified on miniRA3-1 with primers #5 and #13 (RA3 coordinates 1–204; 45429–45909)	*repA-1* allele without *repAp*; intergenic *repA-repB* region
	**pUC18 derivatives**	
pAMB5.2	*repAp,* 256 bp PCR fragment amplified on miniRA3-1 with primers #1 and #4; cloned as the BamHI fragment (RA3 coordinates 45365–45621)	
pAMB5.3	*repAp-1* (IR1*)*,* 256 bp PCR fragment amplified on miniRA3-2 with primers #1 and #4; cloned as the BamHI fragment (RA3 coordinates 45365–45621)	*repAp-1* (IR1*); 3 nt modified in the one arm of the IR1
pAMB5.3.1	*repAp-3* (IR1*), PCR based site-directed mutagenesis of pAMB5.3 with primers #31 and #32	*repAp-3* (IR1*); 6 nt modified in the IR1
pAMB5.2.2	*repAp-2* (IR2*), 256 bp PCR fragment amplified on miniRA3-7 with primers #1 and #4; cloned as the BamHI fragment (RA3 coordinates 45365–45621)	*repAp-2* (IR2*); 5 nt modified in the IR2
pAMB5.19	*repAp repA-1 repBp* [*repXp repX*]*;* 540 bp PCR fragment amplified on miniRA3-1 with primers #1 and #15; cloned as the BamHI fragment (RA3 coordinates 45365–45905)	*repA-1*; mutation A→C at position 45534
pAMB5.25	*repA -repA-1 repBp* [*repXp repX*] *repB’*; 748 bp PCR fragment amplified on miniRA3-1 with primers #1 and #9 cloned; as the BamHI-SphI fragment (RA3 coordinates 1–204; 45365–45909)	*repA-1*; mutation A→C at position 45534
pAMB5.29	*‘repB;* 848 bp EcoRI-SalI fragment from pMOB1.6.1	part of *repB* encoding amino acids from 180 to 459
pAMB5.30	*‘repA repBp* [*repXp repX*] *repB’,* 1kb EcoRI fragment from miniRA3-1 (RA3 coordinates 1–537; 45486–45909)	
pAMB5.30.1	*‘repA repBp* [*repXp-1 repX*] *repB’;* PCR based site- directed mutagenesis of pAMB5.30 with primers #23 and #24 to introduce *repXp-1* mutation	*repXp-1*; A→G in a putative -10 motif of *repXp* (position 45869)
pAMB5.31	*‘repA repBp* [*repXp repX-1*] *repB’;*960 bp EcoRI fragment from pAMB2.4 (RA3 coordinates 1–537; 45486–45909)	*repX-1*; codon ATG for RepX eliminated (mutation A→G at position 45734)
pAMB5.34	*repB* DR2; 1545 bp fragment from miniRA3-1 cloned as the EcoRI-HindIII fragment (RA3 coordinates 537–2082)	
pAMB5.38	pUC18–miniRA3-1 hybrid plasmid; pMOB1.16 digested with EcoRI-PvuII and ligated with pUC18 digested EcoRI-HincII; Ap^R^, Km^R^ (RA3 coordinates 1–2082; 38989–39371; 45486–45909)	*repA-1*; mutation A→C at position 45534
pMOB1.9	*repBp* [*repXp*];365 bp PCR fragment amplified on RA3 with #8 and #9 primers, cloned as the SphI-BamHI fragment (RA3 coordinates 1–204; 45748–45909)	
pMOB1.10	*repAp repA;*376 bp PCR fragment amplified on RA3 with #1 and #2 primers, cloned as BamHI fragment (RA3 coordinates 45365–45741)	
pMOB1.13	r2; 42 nt oligonucleotides #39 and #40 after annealing were cloned between BamHI-SalI sites	
pMOB1.14	r1; 38 nt oligonucleotides #41 and #42 after annealing were cloned between SalI-PstI sites with inactivation of SalI site	
pMOB1.15	r2 r1; 38 nt oligonucleotides #41 and #42 after annealing were cloned between SalI-PstI sites of pMOB1.13 with inactivation of SalI site	
	**pBGS18 derivatives**	
pAMB6.13	truncated DR2; 278 bp PCR fragment amplified on pABB21.1 with primers #22 and #76; cloned as the EcoRI-SacI fragment (RA3 coordinates 1353–1631)	
pAMB6.18	*‘repB* r1; 493 bp fragment from miniRA3-1 inserted as the BglII fragment into BamHI site (RA3 coordinates 1068–1561)	
pAMB6.22	truncated DR2; 556 bp PCR fragment amplified on pABB21.2 with primers #22 and #76; cloned as the EcoRI-SacI fragment (RA3 coordinates 1353–1909)	
pAMB6.24	*‘repB*; 639 bp fragment from pAMB5.29; cloned as the SmaI-HincII fragment (RA3 coordinates 746–1385)	
pAMB6.28	DR2; 729 bp PCR fragment amplified on pABB21 with primers #22 and #76; cloned as the EcoRI-SacI fragment (RA3 coordinates 1353–2082)	
pAMB6.35	*‘repB* DR2; 1336 bp fragment from pAMB5.34 inserted as the HincII-HindIII fragment between SmaI-HindIII sites	
pMOB1.6	*repB*; 1407 bp PCR fragment amplified on RA3 template with the use of #75 and #10 primers; cloned as the SacI-SalI fragment (RA3 coordinates 1–1385; 45887–45909)	
	**pPT01 derivatives**	
pAMB7.2	*repAp*; 256 bp BamHI fragment from pAMB5.2	*repAp-xylE* transcriptional fusion
pAMB7.3.1	*repAp-3* (IR1*); 256 bp BamHI fragment from pAMB5.3.1	*repAp-3* (IR1*)-*xylE* transcriptional fusion; 6 nt modified in the IR1
pAMB7.4	*repAp-2* (IR2*); 256 bp BamHI fragment from pAMB5.4	*repAp-2* (IR2*)-*xylE* transcriptional fusion; 5 nt modified in the IR2
pAMB7.7	*repBp* [*repXp-1*]; 365 bp fragment PCR amplified on pAMB5.30.1 with primers #8 and #9; cloned as the BamHI fragment	*repXp-1-xylE* transcriptional fusion (RA3 coordinates 1–204; 45748–45909)
pAMB7.8R	*repBp* [*repXp-2*]; 365 bp PCR fragment amplified on pAMB2.5 with primers #11 and #13; cloned as the BamHI-SphI fragment	*repB-xylE* transcriptional fusion (RA3 coordinates 1–204; 45748–45909)
pAMB7.11	*repBp* [*repXp*]*;* 391 bp PCR fragment amplified on pJSB18 with primers #18 and #9; cloned as the BamHI-SphI fragment	*repXp-xylE* transcriptional fusion (RA3 coordinates 1–204; 45722–45909)
pAMB7.12	*repBp* [*repXp-1*]; 365 bp PCR fragment amplified on pAMB1.12 with primers #11 and #13, cloned as the BamHI-SphI fragment	*repBp-xylE* transcriptional fusion (RA3 coordinates 1–204; 45748–45909)
pAMB7.14	*repAp repA-1*; 376 bp PCR fragment amplified on miniRA3-1 with primers #1 and *#2*; cloned as the BamHI fragment	*repAp repA*-*1-xylE* transcriptional fusion (RA3 coordinates 45365–45741)
pAMB7.17	*repA repBp* [*repXp repX*]*;* 494 bp PCR fragment amplified on miniRA3-1 with primers #7 and #9; cloned as the BamHI-SphI fragment	*repXp repX-xylE* transcriptional fusion (RA3 coordinates 1–204; 45619–45909)
pAMB7.19R	*repAp repA-1 repBp* [*repXp repX*]*;* 540 bp fragment from pAMB5.19 cloned as the BamHI fragment	*repAp repA-1 repBp* [*repXp repX*]*-xylE* transcriptional fusion (RA3 coordinates 45365–45905)
pAMB7.20	*repAp repA repBp* [*repXp-1 repX*]*;* 540 bp PCR fragment amplified on pAMB1.14 with primers #1 and #15; cloned as the BamHI fragment;	*repAp repA repBp* [*repXp-1 repX*]*-xylE* transcriptional fusion (RA3 coordinates 45365–45905), in the background *repXp-1* mutation (position 45869)
pAMB7.20.1	*repAp repA-1 repBp* [*repXp-1 repX*]*;* PCR-based site-directed mutagenesis of pAMB7.20 with primers #33 and #34 to introduce *repA-1* mutation	*repAp repA-1 repBp* [*repXp-1 repX*]*-xylE* transcriptional fusion, in the background *repXp-1* mutation (position 45869)
pAMB7.21	*repAp repA-1 repBp* [*repXp repX-1*]; 540 bp PCR fragment amplified on miniRA3-4 with primers #1 and #15, cloned as the BamHI fragment	*repAp repA-1 repBp* [*repXp repX-1*]*-xylE* transcriptional fusion, in the background *repX-1* mutation (position 45734)
pAMB7.23	*repA-1 repBp* [*repXp repX*]; 672 bp PCR fragment amplified on miniRA3-1 with primers #5 and #9; cloned as the BamHI-SphI fragment	*repXp repX* [*repA-1 repBp*]*-xylE* transcriptional fusion (RA3 coordinates 1–204; 45441–45909), in the background *repA-1* mutation (position 45534)
pAMB7.39	*repAp repA repBp* [*repXp repX*]*;* 540 bp PCR fragment amplified on pJSB18 (WT mini RA3) with primers #1 and #15, cloned as the BamHI fragment	*repAp repA repBp* [*repXp repX*]*-xylE* transcriptional fusion
pAMB7.39R	*repAp repA repBp* [*repXp repX*]; 540 bp BamHI fragment of pAMB7.39 in the reversed orientation	*repXp repX* [*repAp repA repBp*]*-xylE* transcriptional fusion
pAMB7.39.1	*repAp repA repBp* [*repXp repX-1*]*;* PCR-based site-directed mutagenesis of pAMB7.39 with primers #29 and #30 to introduce *repX-1* mutation	*repAp repA repBp* [*repXp repX-1*]*-xylE* transcriptional fusion, in the background *repX-1* mutation (position 45734)
pMOB1.7.1	*repBp* [*repXp*]*;*365 bp PCR fragment amplified on RA3 with #11 and #13 primers, cloned as the SphI-BamHI fragment (RA3 coordinates 1–204; 45748–45909)	*repBp* [*repXp*]*-xylE* transcriptional fusion
pMOB1.9.1	*repBp* [*repXp*]*;*365 bp SphI-BamHI fragment from pMOB1.9 (RA3 coordinates 1–204; 45748–45909)	*repXp* [*repBp*] *-xylE* transcriptional fusion
pMOB1.10.1	*repAp repA;*376 bp BamHI fragment from pMOB1.10 (RA3 coordinates 1–204; 45365–45741)	*repAp repA-xylE* transcriptional fusion
	**pBBR1MCS-3 derivatives**	
pAMB8.0	pBBR1MCS-3 modified in *tetM* to remove EcoRI site without a change in amino acid sequence of TetM; PCR based site-directed mutagenesis with primers #35 and #36	
pAMB8.36	*repAp repA-1 repBp* [*repXp repX*] *repB*; 2829 bp fragment from pAMB3.36 inserted as the SalI-PstI fragment between XhoI-PstI sites	*repA-1* mutation at position 45534
	**pET28a derivatives**	
pAMB11.41	T7*p*-His_6_-*repA-1*; 296 bp PCR fragment amplified on miniRA3-1 with primers #12 and #3, cloned as the BamHI-SalI fragment (RA3 coordinates 45441–45737)	*repA-1* allele encodes RepAT32P (mutation A→C at position 45534)
pAMB11.42	T7*p*-His_6_-*repA-3*; 300 bp BamHI-SalI fragment from pAMB3.42	*repA-3* allele encodes RepAD76V (mutation A→T at position 45667)
pAMB11.43	T7*p*-His_6_-*repA-4*; 300 bp BamHI-SalI fragment from pAMB3.43	*repA-4* allele encodes RepAL57Q (mutation T→A at position 45610)
pAMB11.44	T7*p*-His_6_-*repA-5*; 300 bp BamHI-SalI fragment from pAMB3.44	*repA-5* allele encodes RepAP9L (mutation C→T at position 45466)
pAMB11.47	T7p-His_6_-*repA*; 296 bp PCR fragment amplified on RA3 with primers #12 and #3, cloned as the BamHI-SalI fragment (RA3 coordinates 45441–45737)	WT RepA

Alleles in brackets [] are encoded on the complementary strand, ‘ denotes gene truncation from the indicated side, * refers to the mutated motif.

**Table 3:** List of primers used in this work.

No(#)	Designation	Sequence
1	ant1	C**GGATCC**GCGGGCCTGATCTATTGTTG
2	ant2	C**GGATCCGCATGC**TTTCTATGCCGCTAACGGC
3	ant2Sal	C**GTCGAC**TATGCCGCTAACGGCCTCAC
4	ant5	CG**GGATCC**TAGCTGCTGCCAGGATAAAC
5	ant6	C**GGATCC**ATGAACCAATCACGACCGGC
6	ant6Eco	C**GAATTC**ATGAACCAATCACGACCGGC
7	ant7	C**GGATCC**CTACACGAACAGAGCCGGAA
8	rep1	C**GGATCC**CCGGAAACCAACTTGGCG
9	rep2	C**GGATCCGCATGC**CGCATAAACTCGGCCTGT
10	rep4	C**CGTCGAC**GCCATCTAAACGGCTTTACA
11	rep5	C**GCATGC**CCCCGGAAAACCAACTTGGCG
12	rep6Bam	C**GGATCC**ATGAACCAATCACGACCGGC
13	rep7	C**GGATCC**GCCGCATAAACTCGGCCTGT
14	rep8	CATGAGCCGGGCTAAATG
15	rep9	C**GGATCCGCATGC**GATGCACCCCTAACTTGCC
16	rep11	GC**GAATTC**ATGGCGCAAGCTCAGTTGTC
17	rep12	**GAATTC**TGCACCCCTAACTTGCCAAGG
18	endrepAF	C**GGATCC**GCCGTTAGCGGCATAGAAAG
19	repAmodF	CT**GAGCTC**GAGGGA**GGATCC**ATGAACCAATCACGACCG
20	1527R2	CACCTTCAGCGGTCGTCAAC
21	orf02pR	GC**GCATGCC**GATCACGCTCCCAGGTCAA
22	terrepBFEco	CC**GAATTCGGTACC**ACAGGCGGCTAGGTGTAAAG
23	mutRep1	GAGCCTGGAT**AAGCTT**AAGGGTTGCACCTCCTATTATGGCGGGAGTGTA
24	mutRep2	GGTACACTCCCGCCATAATAGGAGGTGCAACCCTT**AAGCTT**ATCCAGGC
25	opA1	CTAGGTTACAC**TCTAGA**ACACATCATTCTG
26	opA2	GAATGATGTGT**TCTAGA**GTGTAACCTAGTTG
27	opB1	CTTCAAAACA**GTCGAC**TCTGATGAGGGCTT
28	opB2	GCCCTCATCAGA**GTCGAC**TGTTTTGAAGTG
29	repXm1	GTTAGCGGCGTAGAAAGG**GAGCTC**CCCGGAAACC
30	repXm2	TTCCGGG**GAGCTC**CCTTTCTACGCCGCTAACGG
31	cdMtopA1	CTATTGTTGAAGT**TCGCGA**ACTAGGTTACAC
32	cdMtopA2	GTAACCTAGT**TCGCGA**ACTTCAACAATAGATC
33	wpMt605L	GGCCACCCAT**CCCGGG**TGGACAAGAAAG
34	wpMt605R	CTTTCTTGTCCA**CCCGGG**ATGGGTGGCC
35	modEcTcF	CATGAGAATTGTTGAAGACG
36	modEcTcR	CGTCTTCAACAATTCTCATG
37	mRA3ecoa	GATACTTGAAAGGGAGTTCTTGGCCCCG
38	mRA3ecob	GTACGGGGCCAAGAACTCCCTTTCAAG
39	dr2BamHI	GATCCGCCAAGTTCAGATCTGGACGCCAGAAGGAAATCAACCAGGTGG
40	dr2SalI	TCGACCACCTGGTTGATTTCCTTCTGGCGTCCAGATCTGAACTTGGCG
41	dr1SalI	TCGAAGCCAACTCACCAGGCACCGGCAGCAGCTCGACCAGGTGCTGCA
42	dr1PstI	GCACCTGGTCGAGCTGCTGCCGGTGCCTGGTGAGTTGGCT
43	sphmob	GC**GCATGC**TTTTCTCGTTGGAGGGTGAT
44	inc230P	GC**GGATCC**GATAGCTCTTTGCCATTAAC
45	wt1palG	TTGAAGTTTACCAACTAGGTTACACTTCAA
46	wt1palD	TTGAAGTGTAACCTAGTTGGTAAACTTCAA
47	wt2palG	AAACACATCATTCTGATGAGGGC
48	wt2palD	GCCCTCATCAGAATGATGTGTTT
49	mutAll1palG	TTGAAGT**TCGCGA**ACTAGGTTACACTCTAG
50	mutAll1palD	CTAGAGTGTAACCTAGT**TCGCGA**ACTTCAA
51	mutAll2palG	AAACA**GTCGAC**TCTGATGAGGGC
52	mutAll2palD	GCCCTCATCAGA**GTCGAC**TGTTT
53	repB2F	CATCGAGAAGCAAAAGGCG
54	repB2R	CCAACTTGCGTAGGTCTTCCAG
55	galK-F	ATGATCTTTCTTGCCGAGCG
56	galK-R	AGCAGCTTTATCATCTGCCGC
57	repAF	CAAACAGACTTGGCCACCC
58	repAR	GACTGTAACAGGCACTCGCC
59	repAF1	GATAGCGCGTTTATCCTGGC
60	repAR1	CTCGTCATTCTCTGCGTCCC
61	repBF	CTGGAATGCTTGCCAAACCC
62	repBR	TTCACGGTATTGACCAGGCG
63	repBR1	TCACTTTGAAATAGCCATCTAAACGG
64	02prevUF	TGTAAAGCCGTTTAGATGGC
65	02prevUR	CAGCATGGCTATACGCCTGC
66	02prevUF1	GTTAGCAGGCGTATAGCCATG
67	02prevUR1	CTGGCAAGTTGATCTAAAGG
68	r2F	GTTCAGATCTGGACGCCAGAAG
69	02prevDF	GTACGAAATCAGGCGACGCTATGC
70	02prevDR	GGCAATAAAAAGCGCGCTCTAC
71	EcoOrf2F	CG**GAATTC**ATGATCCACACAGCTAACCG
72	SalOrf2R	CGC**GTCGAC**ATAGGCCAAATCGGCCTACT
73	EcrepB1	ACCGTCAACTCAAAGAGCGG
74	EcrepB2	CCGCTCTTTGAGTTGACGGT
75	rep3	CC**GAGCTC**TTGGCAAGTTAGGGGTGCAT
76	CmR	**GAGCTC**GCCCGGTAGTGATCTTATTTC
77	NorRepBF	TAACTTTCTCCTTCTCTCTGG
78	NorRepB7	GAAT*TAATACGACTCACTATAGGG*TAGTAATAGGGGAGGGACTTG

Restriction sites introduced in the primers are in bold, the modified nucleotides are underlined, and the T7p sequence is in italics.

RNA isolation and analysis

- RT reaction followed by PCR

Overnight *E. coli* DH5α(RA3) and DH5α(WT miniRA3) cultures were diluted 1:100 into fresh L broth supplemented with chloramphenicol and tetracycline, respectively, and propagated with shaking until the optical density at 600 nm (OD600) reached 0.4 to 0.6. A total of 2 mL samples were mixed with 4 mL of RNAprotect Bacteria reagent (Qiagen). Total RNA was isolated using the RNeasy minikit (Qiagen), according to the manufacturer’s instruction, and treated with the Turbo DNase kit (Ambion) to remove DNA contamination. The control PCRs were conducted on purified RNA to ensure lack of a DNA template. The RNA concentration was estimated using the NanoDrop ND-1000 spectrophotometer (Thermo Fisher Sci PL). The integrity and overall quality of RNA preparation were assessed by native agarose gel electrophoresis [31]. cDNA synthesis and purification were performed with the 5′RACE System for Rapid Amplification of cDNA Ends, version 2.0 (Invitrogen). Briefly, 4 µg of total RNA were used per reaction with SuperScript II reverse transcriptase (RT) and primers #57, #63, #64, and #66, respectively (Table 3). The bulk of RNA was removed with a mixture of RNases T1 and H. cDNA was then used as a template for PCRs with appropriate pairs of primers. PCR products were analyzed by agarose gel electrophoresis; gels were stained with ethidium bromide and photographed.

- Northern analysis

Total RNA was purified with a commercially available kit (Qiagen) from *E. coli* DH5α strain carrying RA3, WT miniRA3, or miniRA3 mutant derivatives. A total of 0.5–12 μg of the total RNA was denatured and separated on the denaturing agarose gels with the RNA standards treated the same way [31]. The track with RNA standards was cut off, stained with the ethidium bromide, and photographed with a ruler to facilitate sizing of detected mRNAs. The gels were then treated in two different ways depending on the probe used.

(a) Hybridization with the radioactive single stranded DNA labelled at 5′ end as a probe

The gels were rinsed with DEPC-treated water and incubated for 20 min in the transfer buffer (3 M NaCl, 0.01 M NaOH). The RNA samples were transferred on a nitrocellulose membrane by 3–4 h using a vacuum pump. The RNA was cross-linked to the membrane for 1 min under the UV light.

To prepare a radioactive probe by PCR the primers #1, #2, and #14 were radioactively labelled by [γ-32P] ATP at the 5′ end with the use of T4 Polynucleotide Kinase according to the instructions. The labelled oligonucleotides were precipitated in the presence of 0.004 μg tRNA (10 μg mL^−1^), 1/6 volume of 3 M sodium acetate (pH 5.2), and 3 volumes of 96% ice-cold ethanol for 24 h at −20 °C. After centrifugation, the pellet was re-suspended in 20 μL of water.

The labelled primer (*) was used in a pair with an unlabelled one in the standard PCR reaction (the number of cycles was increased to 35). For detection of the *repA* transcripts, the 376 bp fragment amplified with #1 and #2* primers was used as a probe, the same fragment amplified with #1* and #2 served as a probe to visualize the *repX* transcripts. To obtain the *orf02prev* probe, a 1169 bp fragment was amplified with the use of primers #14* and #21. The PCR products were purified on a column (QIAquick PCR Purification Kit), denatured for 5 min at 100 °C, chilled on ice for another 5 min, and used for the hybridization. After overnight hybridization, the membranes were washed thoroughly, loaded to the cassette, and analyzed using Phosphorimager (FUJIFILM FLA–7000).

(b) Hybridization with [α– ^32^P] UTP labelled mRNA

After RNA separation, the gel was soaked for 10 min in DEPC treated water, then incubated with 75 mM NaOH for 20 min, followed by incubation for 15 min in 1.5 M NaCl, 0.5 M Tris-HCl (pH 7.4) solution, and soaking in 6× SSC for 20 min. The RNA samples were transferred overnight to a nitrocellulose membrane via upward capillary transfer method [31]. The air-dried membrane was exposed to UV light to cross-link RNA. The fixed membrane was then used in the hybridization with the specific probes, [α– ^32^P] UTP labelled mRNA fragments.

To detect *repB*mRNAs the 485 bp fragment from the 3′ end of the *repB* was PCR amplified with #77 and #78 primers in such a way that #78 primer inserted *T7p* sequence upstream of the complementary strand to *repB*mRNA. The PCR products were purified using QIAquick PCR Purification Kit (Qiagen) and served as templates in the transcription in vitro with T7 RNAP (Fermentas). The reaction mixture contained 0.7–1 μg of the PCR template, 1x transcription buffer, 0.5 mM rNTPs mix (ATP, CTP, and GTP), 12 μM UTP, 50 mCi of [α– ^32^P] UTP and 2 U of T7 RNAP in the final volume of 20 μL. After 2 h of incubation at 37 °C, 2 μL of DNase were added and incubated at 37 °C for 15 min to remove the template DNA. The transcription products were then separated from free [α– 32P] UTP with the use of PCA mixture (phenol/chloroform/isoamyl alcohol in ratio: 25/24/1). After overnight hybridization, the membranes were washed thoroughly, loaded to the cassette, and analyzed using Phosphorimager (FUJIFILM FLA–7000).

In vitro analysis of protein-DNA interactions by electrophoretic mobility shift assay (EMSA)

The reactions were set in a final volume of 20 μL and contained 30 ng of purified PCR fragments (the assay performed on the 0.8% agarose gels) or 150 ng of ds oligonucleotides annealed before the binding (the assay performed on the 10% native polyacrylamide gels). The DNA-RepA (0–100 pmoles of protein) binding reactions proceeded in the binding buffer (25 mM Tris-HCl pH 8.0, 5 mM MgCl_2_, 40 mM NaCl, 0.5 mg mL^−1^ BSA) for 30 min at 37 °C. A total of 10% glycerol was added to the samples prior to the loading on a gel. The gels were run in 0.5×TBE buffer [31]. The gels were stained in ethidium bromide solution, washed, and photographed.

Immunodetection of proteins–western blot analysis

His_6_-tagged proteins separated by SDS-PAGE were transferred onto a nitrocellulose membrane and visualized using mouse anti-His_6_-tag antibodies (Pierce). The membrane was washed and incubated with the secondary anti-mouse antibodies (Promega) (diluted 1:10,000). The development step was carried out in the dark in 20 mL of AP buffer containing BCIP (5-bromo-4-chloro-3-indolyl-phosphate) and 0.04 M NBT (nitro blue tetrazolium) reagents (Promega).

## Figures and Tables

**Table 1:** Stability and copy number of RA3 and miniRA3 variants in various hosts.

Plasmid	Mutations in the Minireplicon	Stability (%) after 60 Generations of Growth without Selection	Plasmid Copy Number per Chromosome (Real-Time qPCR)
		in the *E. coli* DH5α strain	
**RA3**		100%	1
**WT miniRA3**		100%	1
**miniRA3-1**	*repA-1 (RepAT32P)*	100%	2.1
**miniRA3-2**	repAp-1 (IR1*) repA-1	100%	74.6
**miniRA3-7**	*repAp*-*2* (IR2*) *repA*-*1*	100%	43.3
**miniRA3-4**	*repX-1, repA-1*	100%	13.8
**miniRA3-5**	*repXp-1*	100%	2.2
		in the *P. putida* KT2440 strain	
**RA3**		10% (±3)	1
**WT miniRA3**		<3% *	1
**miniRA3-1**	*repA*-*1* (RepAT32P)	31% (±4)	4.6
**miniRA3-2**	*repAp*-*1* (IR1*) *repA*-*1*	12% (±8)	7.6
**miniRA3-7**	repAp-2 (IR2*) repA-1	18% (±3)	9.8
**miniRA3-4**	*repX-1, repA-1*	27% (±4)	3.6

* Plasmid retention after 20 generations of growth without selection.

## Data Availability

All obtained data is included in the manuscript and Supplementary Materials.

## Supplementary Materials

The following supporting information can be downloaded at: https://www.mdpi.com/article/10.3390/ijms23179964/s1.
