# Supplementary material for: Deciphering the Regulatory Circuits of RA3 Replication Module - Mechanisms of the Copy Number Control
Source: Int J Mol Sci. 2022 Sep 1;23(17):9964. doi: 10.3390/ijms23179964 (PMC9455977; doi:10.3390/ijms23179964)
Supplement: Supplementary file 1 [file ijms-23-09964-s001.zip › FigureS1.pdf]

```
RA3        MAQ-AQLSLFGQ-NEAETYHDTSARGFFSLLVQWP--TGKRQSSFPLEQMPTVLNAVDHRLDTWLSQAEFMRPNRRVVNLLRIGLLFADLDTYKMPWAQGRKPEE 101
Rms149     MGQ-AQLRLFSPADEAGCYHDTARQGFFSLLMATGEGSGKKQDSYRLSQMPVVLSMLDHSRDTWLSQAEFIKPNRRVVNLARIGLLFADLDTYREPWAQGRSPEQ 104
Plasmid2   MAADQQLSLFDRDEEARAYHDPEQAGFFSILVNAK--GDKRQSSHRLIDMPTVLGLIDKNRDTWMTQAEFIRPNRRVVNLARVGLLFADLDTYRKPWASGRTPEQ 103
pAMTVIM-7  MAGQAQLALFEPADEAGTYHDTARTGFFSLLVAVG--KDKRQDSYRLTDMPTILGMVDQTRDTWLTQAEFMRPNRRVVNLARVGLLFADLDTYRQPWATGRTPEQ 103
pRSB105    MGQ-AQLRLFSPADEAGCYHDTARQGFFSLLMATGEGSGKKQDSYRLSQMPVVLSMLDHSRDTWLSQAEFIKPNRRVVNLARIGLLFADLDTYREPWAQGRSPEQ 104
pRIO-5     MGQ-AQLRLFSPADEAGCYHDTARQGFFSLLMATGEGSGKKQDSYRLSQMPVVLSMLDHSRDTWLSQAEFIKPNRRVVNLARIGLLFADLDTYREPWAQGRSPEQ 104
pAb5S9     MAAVEQLSLFDRAEEARAYHDPARAGFFSILVDVN--GDKRQSSHRLIEMPTVLELIDKSRDTWLTQAEFIRPNRRVVNLARLGLLFADLDTYRTPWAKGRTPEQ 103
pKBB4037   MAAVAQLSLFDRAEEARAYHDPGRSGFFSILVDVH--GDKRQSSHRLVEMPTVLELIDKTRDTWLTQAEFIRPNRRVVNLARIGLMFADLDTYRQPWATGRTPEQ 103

RA3        LAAAITYFCQSEGIPQPSIMVYSGRGIQAKWLLERPVPRQALPRWNACQTHLINKLAELGADPGAKDASRVLRLVNTVNSKSGAVCRVVHVLEE-GGLPIRYNFE 205
Rms149     LAAAVMFRCYDEGVPPPSILVFSGRGVQAKWLLDGTLPRQALPRWNACQRYLIDRLAGLGADPAAKDASRVLRLVNTVNSKSGEVCRVIHVEQGPDGEPIRYNFE 209
Plasmid2   LVDSVLYHCTQEGIPPPSILVFSGRGIQAKWLLEGTLPRQALPRWNACQRYLIDRLAYLGADRQAKDASRVLRVVQTVNEKSGEICRVVHVQNELNGQPVRYNFE 208
pAMTVIM-7  LAATVLYHCAQEGIPTPSLLVYSGRGIQAKWLLDGTLPRQALPRWNACQRYLVDRLAGLGADPQAKDASRVLRLVSTVNTKSGDVCRVVHVEHGQDGQPVRYGFE 208
pRSB105    LAAAVMFRCYDEGVPPPSILVFSGRGVQAKWLLDGTLPRQALPRWNACQRYLIDRLAGLGADPAAKDASRVLRLVNTVNSKSGEVCRVIHVEQGPDGEPIRYNFE 209
pRIO-5     LAAAVMFRCYDEGVPPPSILVFSGRGVQAKWLLDGTLPRQALPRWNACQRYLIDRLAGLGADPAAKDASRVLRLVNTVNSKSGEVCRVIHVEQGPDGEPIRYNFE 209
pAb5S9     LAASVLYHCAQEGIPTPSMLIFSGRGIQAKWLLDGTIPRQALPRWNACQKYLIDRLRPVGADVAAKDASRVLRLVETVNSKSGEICRVVHVENGPDGQPVRYSFE 208
pKBB4037   LAASVLYHCAQEGIPTPSLLIFSGRGIQAKWLLDRPVPRQALPRWNACQKYLIDRLMAIGADVAAKDASRVLRLVDTVNTKSGEICRVVHVETGADGQPVRYGFE 208

RA3        YLAEMLLPVARWDIE--KQ-KAERQ-QARLTVVNGGKVGNLRTFSGRQLAWDRLEDLRKLGQLRGGINEGERMRHLFWRLNFLLLSGATNSAQMYHEAAALSSEL 306
Rms149     YLAEALLPVARWDIEADRKARAD---RRQFKLLPGGQTGNLRTLNGRQLAWDRLEDLRTLAALRGGVAEGERMQHLFWRLNFLLLSGATHTGQMYHEAAALAREL 311
Plasmid2   YMAEILLPVARWTIEQQRQEREKRAIRRQLKLLPGDKTDNLRGFSGRQLAWHRLEDLRTLAELRGGVQEGERMQHLFWRLNFLLLSGATNSRLMYYEAKALTKEL 313
pAMTVIM-7  YLAEMLLPVARWDIEQQRRDRAE---RRQLKLLPGGKADNLRGFSGRQLAWDRLEDLRKLGELRGGVREGERMQHLFWRLNFLLLSGATHSGQMYHEAAALAGEL 310
pRSB105    YLAEALLPVARWDIEADRKARAD---RRQFKLLPGGQTGNLRTLNGRQLAWDRLEDLRTLAALRGGVAEGERMQHLFWRLNFLLLSGATHTGQMYHEAAALAREL 311
pRIO-5     YLAEALLPVARWDIEADRKARAD---RRQFKLLPGGQTGNLRTLNGRQLAWDRLEDLRTLAALRGGVAEGERMQHLFWRLNFLLLSGATHTGQMYHEAAALAREL 311
pAb5S9     YLAEMLLPVARWTIEQQRQERAE---RRQLKLLSGAKTDNLRGFSGRQLAWHRLEDLRKLATLRGGVSEGERMQHLFWRLNFLLLSGATHSSQMYYEAKALAGEL 310
pKBB4037   YLAEMLLPVARWEIEQQREARAER--RQQLKLLPGTKTDNLRGFSGRQLAWHRLEDLRKLATLRGGVQEGERMQHLFWRLNFLLLSGATHSGQMYHEAKALAREL 311

RA3        DTRWSYRSKELMTLYSKAKSYEAGERVEFGGKSFAPLYTPKNDTLINLFQISQQEQEQLKTIISTDEAQKRRRERDRLRDEERRRAAGQ----LEREAYEANSLS 407
Rms149     DPRWNYRSAELMTLYAKAKAHEAGEKVEFGGKQFAPLYTPKNDTLISLFHISDDEQRKLRTLISRDMATERRRDRDRKRDEARRRAAGA----VDRATYEANSAS 412
Plasmid2   DPNWQENSKELMTLYSKAKAYEAGEKVSFGGREYAPLYTPRNDTLINLFQITDDEQAQLRTIISRDMAKERDRAR----KEAVRRAAGA----VDRETYESQSLS 410
pAMTVIM-7  DPAWSYRSKELMTLYAKAKAYEAGERVTLGGREFAPLYTPKNDTLISLFQITDDEQRKLRTLISSDMAKERDRER----HTARRRAAGA----VDREYYLDAAEA 407
pRSB105    DPRWNYRSAELMTLYAKAKAHEAGEKVEFGGKQFAPLYTPKNDTLISLFHITDDEQRKLRTLISRDMAAERHSER----EKARRRAAGA----VDRASYLEAASA 408
pRIO-5     DPRWNYRSAELMTLYAKAKAHEAGEKVEFGGKQFAPLYTPKNDTLISLFHITDDEQRKLRTLISRDMAAERHSER----EKARRRAAGA----VDRASYLEAASA 408
pAb5S9     APTWAYRDKELMTLYSKAKAYEKGEKISFAGREFAPLYTPKNDTLINLFQITDDEQAQLRTIISKGMAKGRDRAR----KEAARRAAGA----VDRETYLEAANA 407
pKBB4037   APDWQQNSKELMTLYSKAKAYEAGEKITFGGKEYAPLYTPRNDTLINLFQITDQEQCELRTIISRDMAAERRRSA----TESATRPVGGPLVRWIGKTYLEAANT 412
             HTH
RA3        RQKPWEAMGMSRAKWYRLGKPSPQQNSETSPSPITNGEASAPPLQAARCKAV----------------------------------------------------- 459
Rms149     RQKPWESLGMSRASWYRAGKPMPA--CETGSSPITASKVERKA-------------------------------------------------------------- 453
Plasmid2   RQKPWEALGMSRASWYRAGKPMPA-NSETSPSVLQKGNGRFLV-------------------------------------------------------------- 452
pAMTVIM-7  KRAQAQALKAEGLSVRAIAQRMGISKSLAAMYAKEAAECP-------------KSVR----ITADQAMPVASIAGSDEGGREGVQSPSVLIMAEPTGRGAV---- 491
pRSB105    KQAQALALKAQGLSVRAIAAQMGISKTAAGRYIAEPGECP-------------KSMR----ITGGEG-------------------------------------- 458
pRIO-5     KQAQALALKAQGLSVRAIAAQMGISKTAAGRYIAEPGECP-------------KSMR----ITGGEG-------------------------------------- 458
pAb5S9     KQAQAQALRAQGLSIRAIAQQMGVSVGSVSGYLKAAPGVQSPSVLQADLAGCSKSVR----ITNGEAHARPVGPAFE---------------------------- 480
pKBB4037   KQAQAQALREQGLSVRAIAAQMGVSVGSVSGYLKAGASVQSGSPITGAVMEKSG-VQSPSPITNGEAYARPVGPAFE---------------------------- 488
```

Figure S1. Comparison of RepB homologs. Residues identical and similar in at least four proteins are shadowed in dark grey or light grey, respectively. The red arrow marks the putative Helix-Turn-Helix motif in RepB of RA3. RA3 (ABD64829); Rms149 of *Pseudomonas aeruginosa* (WP_011270176); Plasmid 2 of *Nitrosomonas eutropha* (WP_011630685); pAMTVIM-7 of *P. aeruginosa* (WP_011997473); pRSB105 (ABI20460); pRIO-5 of *Serratia marcescens* (WP_015060213); pAb5S9 of *Aeromonas bestarium* (WP_011931113); pKBB4037 of *Bordetella bronchiseptica* (CAI47016).
