# Supplementary material for: Deciphering the Regulatory Circuits of RA3 Replication Module - Mechanisms of the Copy Number Control
Source: Int J Mol Sci. 2022 Sep 1;23(17):9964. doi: 10.3390/ijms23179964 (PMC9455977; doi:10.3390/ijms23179964)
Supplement: Supplementary file 1 [file ijms-23-09964-s001.zip › FigureS2.pdf]

Fragment 45619-45835:

```
#7
CTACACGAACAGAGCCGGAATACTGGCTAGGGCTGCAAAGTGCCGTGGATCTATGGGACG
GATGTGCTTGTCTCGGCCTTATGACCGATCCCGACGTTTCACGGCACCTAGATACCCTGC
 a  t  r  t  e  p  e  y  w  l  g  l  q  s  a  v  d  l  w  d
-  v  f  l  a  p  i  s  a  l  a  a  f  h  r  p  d  i  p  r

                                                  #18
CAGAGAATGACGAGGCGTTGCGTAGTCGTCTAGGACAGGTGAGGCCGTTAGCGGCATAGA
GTCTCTTACTGCTCCGCAACGCATCAGCAGATCCTGTCCACTCCGGCAATCGCCGTATCT
 a  e  n  d  e  a  l  r  s  r  l  g  q  v  r  p  l  a  a  -
l  s  h  r  p  t  a  y  d  d  l  v  p  s  a  t  l  p  m

          #8          -35 repBp              -10 repBp   RepX
AAGGGAAAACCCCGGAAACCAACTTGGCGGGCGGTTTACCGGGGTCTAAATTTGGTGGCA
TTCCCTTTTGGGGCCTTTGGTTGAACCGCCCGCCAAATGGCCCCAGATTTAAACCACCGT
#2

CTTTCGGTGAGCTTGGCGGCAACCGAGGTGCCGGAGC
GAAAGCCACTCGAACCGCCGTTGGCTCCACGGCCTCG
```

Figure S2. DNA sequence of the 3' end of the *repA* gene, *repX* ORF and *repBp*
The top strand encodes the C-terminus of RepA (in red), the bottom strand encodes 38 amino acids polypeptide RepX (in green). The rbs site of *repX*mRNA is underlined. Arms of the palindromic sequence are shadowed in blue, T-rich sequences on both sides of the palindrome are underlined also in blue. Putative *repBp* motifs are in bold. Primers used for cloning of various derivatives are shown.
