# Supplementary material for: Deciphering the Regulatory Circuits of RA3 Replication Module - Mechanisms of the Copy Number Control
Source: Int J Mol Sci. 2022 Sep 1;23(17):9964. doi: 10.3390/ijms23179964 (PMC9455977; doi:10.3390/ijms23179964)
Supplement: Supplementary file 1 [file ijms-23-09964-s001.zip › FigureS3.pdf]

A

B

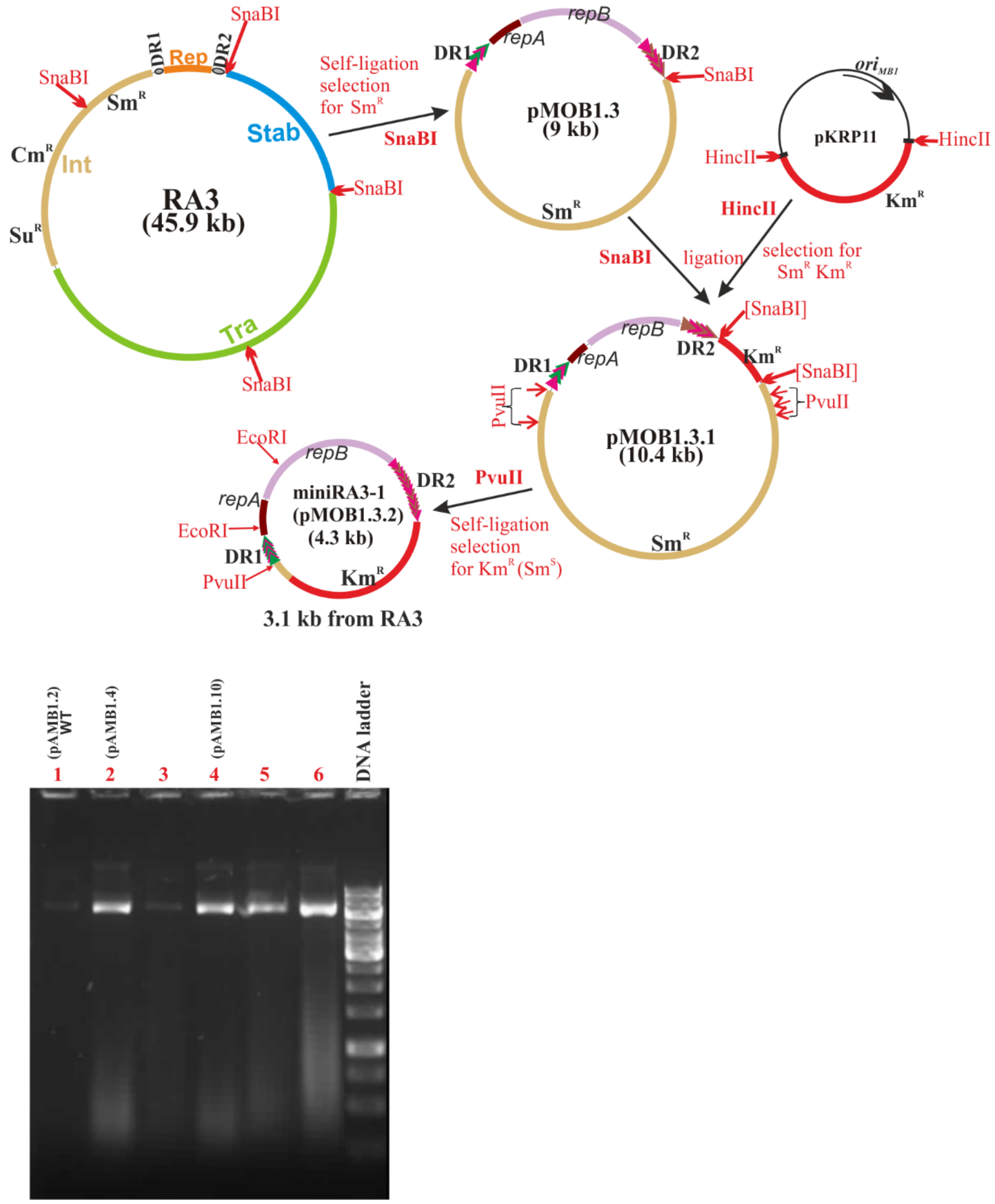

Figure S3. RA3 minireplicons construction

(**A**) The restriction sites used in subsequent steps of construction are indicated. The identified elements of replication module as well as the resistance cassettes are shown. (**B**) Plasmid DNAs isolated from the *E. coli* Sm^R colonies obtained after transformation with RA3 plasmid SnaBI digested and then self-ligated. Plasmid numbers refer to the isolates that have been further analyzed by sequencing.
