# Supplementary material for: Deciphering the Regulatory Circuits of RA3 Replication Module - Mechanisms of the Copy Number Control
Source: Int J Mol Sci. 2022 Sep 1;23(17):9964. doi: 10.3390/ijms23179964 (PMC9455977; doi:10.3390/ijms23179964)
Supplement: Supplementary file 1 [file ijms-23-09964-s001.zip › FigureS4.pdf]

Fragment between 1291-1551 nt:

```
           partial r3
CAACAAAACAGTGAGACAAGTCCCTCCCCTATTACTAATGGCGAAGCCTCAGCCCCCCCG
GTTGTTTTGTCACTCTGTTCAGGGAGGGGATAATGATTACCGCTTCGGAGTCGGGGGGGC
  q  q  n  s  e  t  s  p  s  p  i  t  n  g  e  a  s  a  p  p
                       #64                    AT-rich region
#22
CTACAGGCGGCTAGGTGTAAAGCCGTTTAGATGGCTATTTCAAAGTGAGTGAGATTTTGT
GATGTCCGCCGATCCACATTTCGGCAAATCTACCGATAAAGTTTCACTCACTCTAAAACA
                                            #10       #63
  l  q  a  a  r  c  k  a  v  -

                                             #66
AACTGATGGCAGCCCTAGAGGCTGCTTTCTTTTGAGGGGTTAGCAGGCGTATAGCCATGC
TTGACTACCGTCGGGATCTCCGACGAAAGAAAACTCCCCAATCGTCCGCATATCGGTACG

                                                  r1
TGGATCTCAGTCTATCACTACCCGCTTAATCTTCCACCTTTAGATCAACTTGCCAGGCAC
ACCTAGAGTCAGATAGTGATGGGCGAATTAGAAGGTGGAAATCTAGTTGAACGGTCCGTG
   #65

                     r2'        BglII
CGGCAGCAGTTCGACCAGGTAGCCAAGTTCAGATCT
GCCGTCGTCAAGCTGGTCCATCGGTTCAAGTCTAGA
```

Figure S4. DNA sequence of *oriV*RA3

The partial r3 repetition is indicated in green, AT-rich patch (60%) in blue and r1 of DR2 and part of r2 (to BglII site in italics) are marked in red and purple, respectively. Underlined nucleotides in the repetition r1 differ from the consensus. Primers used for cloning and RT-PCR are indicated. The amino acid sequence of C-terminus of RepB is shown.
