# Supplementary material for: Deciphering the Regulatory Circuits of RA3 Replication Module - Mechanisms of the Copy Number Control
Source: Int J Mol Sci. 2022 Sep 1;23(17):9964. doi: 10.3390/ijms23179964 (PMC9455977; doi:10.3390/ijms23179964)
Supplement: Supplementary file 1 [file ijms-23-09964-s001.zip › FigureS5.pdf]

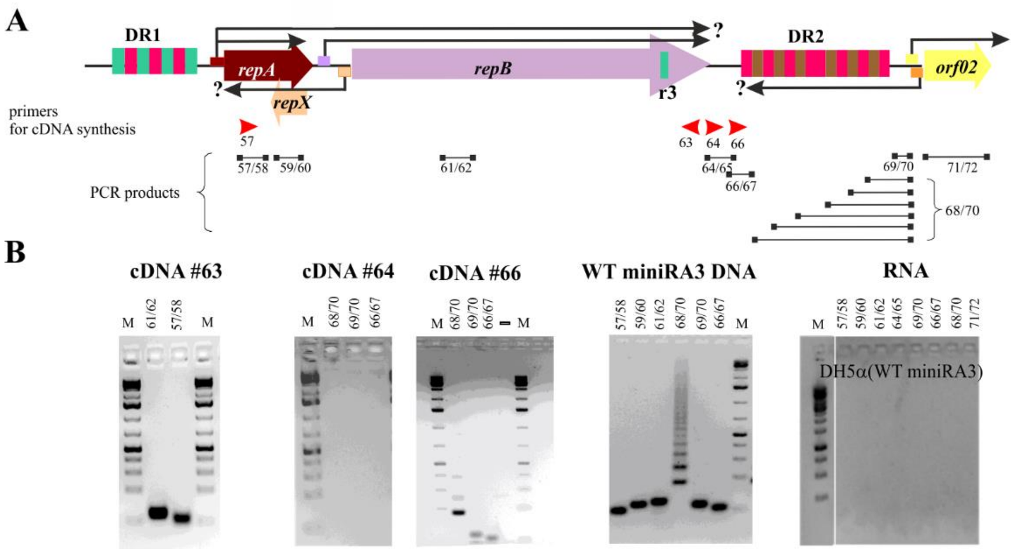

Figure S5.RT-PCR analysis of the transcripts of WT miniRA3. on RNA isolated from DH5α(RA3). The upper part (**A**) illustrates the location of primers used for cDNA synthesis (red arrows) and the expected PCR products for various primers pairs as in the Figure 4. (**B**) Photographs document products of PCR using cDNAs obtained in the reverse transcriptase reactions on RNA isolated from *E. coli* strain DH5a (WT miniRA3) with primers #63 (for the *repA-repB* co-expression), primers #66 and #64. (for the extent of the transcript from *orf02p*$_{rev}$). The last two photographs document control reactions of the used pairs of primers on plasmid DNA and purity of RNA samples. M- indicates DNA markers.
