# Supplementary material for: Deciphering the Regulatory Circuits of RA3 Replication Module - Mechanisms of the Copy Number Control
Source: Int J Mol Sci. 2022 Sep 1;23(17):9964. doi: 10.3390/ijms23179964 (PMC9455977; doi:10.3390/ijms23179964)
Supplement: Supplementary file 1 [file ijms-23-09964-s001.zip › FigureS6final.pdf]

Fragment 45361-45500:

```
                                  (IR1*)                    (IR2*)
   #1                      c   g           c t  g       g t c g  c
CAGGGCGGGCCTGATCTATTGTTGAAGTTTACCAACTAGGTTACACTTCAAAACACATCAT
GTCCCGCCCGGACTAGATAACAACTTCAAATGGTTGATCCAATGTGAAGTTTTGTGTAGTA

                 #6
TCTGATGAGGGCTTATTACATGAACCAATCACGACCGGCCACACCTGGCCAGATACTTGAA
AGACTACTCCCGAATAATGTACTTGGTTAGTGCTGGCCGGTGTGGACCGGTCTATGAACTT
                    m  n  q  s  r  p  a  t  p  g  q  i  l  e

    EcoRI
AGGGAATTCTTGGCCCCG
TCCCTTAAGAACCGGGGC
 r  e  f  l  a  p
```

Figure S6. DNA sequence of the *repA* promoter region
The -35 and -10 motifs of *repAp* are bolded. The rbs site of *repA*mRNA is underlined and the N-terminal sequence of RepA is shown. The arms of two palindromic sequences, IR1 and IR2, are labelled in green (operator O_{RepA}) and in gray (unknown function), respectively. Lowercase letters above the DNA sequence indicate substitutions in IR1* (6 nt) and IR2* (5 nt), introduced to diminish the reverse complementarity of IR1 or IR2 motifs. Restriction site EcoRI (italics) and primers used for PCR amplification are indicated.
